# Unsupervised Multi-Sensor Condition Monitoring of AODD Pump Systems Using Physics-Informed Health Indices and Gaussian Mixture Models

**DOI:** 10.3390/s26165204

**Published:** 2026-08-17

**Authors:** Seong-Wook Kim, Akeem Bayo Kareem, Jang-Wook Hur

**Affiliations:** Department of Mechanical Engineering (Department of Aeronautics, Mechanical and Electronic Convergence Engineering), Kumoh National Institute of Technology, 61 Daehak-ro, Gumi-si 39177, Gyeongsangbuk-do, Republic of Korea; 1rlatjddnr@kumoh.ac.kr (S.-W.K.); 20216004@kumoh.ac.kr (A.B.K.)

**Keywords:** AODD pump, condition monitoring, physics-informed machine learning, Gaussian mixture model, sensor fusion, explainable AI, unsupervised anomaly detection, predictive maintenance, prognostics and health management, sludge transfer

## Abstract

Air-operated double-diaphragm (AODD) pumps in industrial sludge transfer suffer from gradual performance degradation due to rheological variations and component wear, yet conventional monitoring relies on scarce labeled fault data. This paper presents an unsupervised multi-sensor framework that requires no fault labels, integrating physics-informed dual health indices, HI-P (sludge load) and HI-V (mechanical stress), with a Gaussian Mixture Model anomaly detector and a physics residual attribution module. Governing equations motivate the use of these indices from five sensors: inlet and outlet flow meters (100 Hz), an air pressure transducer (100 Hz), and inlet and outlet accelerometers (1652 Hz). Trained on one healthy baseline day (86,218 one-second windows), the Gaussian Mixture Model achieves 100% day-level classification performance on the evaluated dataset (F1 = 1.00) across 455,201 test windows from nine operating days, with window-level receiver operating characteristic area under the curve (ROC-AUC) = 0.8580 and precision–recall AUC (PR-AUC) = 0.9082. Residual attribution analytically confirms that pressure residuals drive Episode 1 (HI-P peak 3.63 times baseline, Cohen’s d = 1.70) and vibration residuals drive Episode 2 (HI-V peak 5.44 times the baseline, d = 4.10), providing empirical support for the proposed physics-informed formulation without requiring fault labels. Comparisons with four unsupervised benchmarks confirm that this is the only approach that simultaneously enables label-free operation, physics-driven features, exact attribution, real-world deployment, and perfect day-level F1.

## 1. Introduction

Air-operated double diaphragm (AODD) pumps are widely deployed in wastewater treatment, mining, and chemical processing for transferring abrasive, viscous, and particle-laden sludge [1,2]. Their compressed-air drive and self-priming capability make them well-suited for hazardous and difficult-to-access installations.

AODD pump systems, comprising the pump body together with peripheral components such as filter presses, ball check valves, manifolds, and piping, are susceptible to progressive performance degradation due to changes in sludge rheological properties during long-term operation. As sludge solids concentration increases, its apparent viscosity and yield stress increase, resulting in greater hydraulic resistance, increased pumping energy requirements, and higher mechanical loading on pump components and associated flow paths [3,4]. These changes reduce pumping efficiency and, if left undetected, can lead to unplanned downtime and accelerated component wear.

A critical challenge in industrial condition monitoring is the scarcity of labeled fault data [5,6]. In practical deployments, degradation events are rarely recorded systematically, and the precise onset of deterioration is generally unknown. Furthermore, in AODD pump installations, degradation frequently originates from peripheral components (e.g., clogged filters, worn check valves, and scale accumulation) rather than diaphragm failure itself [7]. These degradation modes produce coupled changes in pressure, flow, and vibration measurements, requiring both multi-sensor fusion and the design of physically interpretable features.

Despite recent advances in machine learning and physics-informed condition monitoring, no existing framework simultaneously combines physics-derived multi-sensor health indices, unsupervised anomaly detection, and physically interpretable anomaly attribution for industrial AODD pump systems operating under variable sludge conditions. This motivates the framework proposed in this paper. This paper makes five principal contributions:

1. We derive governing equations relating sludge effective viscosity to air supply pressure, stroke efficiency, and outlet vibration impact energy and use them to select physically interpretable features from five heterogeneous sensors operating at two native sampling rates.
2. We propose HI-P (sludge load index) and HI-V (mechanical stress index) as complementary, physics-informed health indicators that capture distinct system-level degradation mechanisms.
3. A Gaussian Mixture Model trained exclusively on one healthy baseline day achieves complete day-level identification of degradation episodes (accuracy, precision, recall, F1-score, and specificity: all 100%) across 455,201 one-second windows collected over nine operating days.
4. We introduce a physics-residual attribution framework that analytically decomposes anomaly scores into physically meaningful residual contributions, enabling direct interpretation of the underlying degradation mechanism.
5. We conduct a comprehensive sensor ablation study and benchmark against five unsupervised anomaly detection methods, demonstrating that full three-sensor fusion is required to detect complete degradation episodes.

The remainder of this paper presents the related work, the proposed physics-informed monitoring framework, the experimental evaluation, discussion of the results, and concluding remarks.

## 2. Related Work

This section reviews three interconnected areas, signal-based pump condition monitoring, unsupervised anomaly detection, and explainable AI for condition monitoring, with emphasis on the specific challenges of AODD pump systems in sludge transfer applications.

### 2.1. Signal-Based Methods and Multi-Sensor Fusion for Pump Monitoring

Vibration analysis remains the most widely adopted technique for pump condition monitoring because of its sensitivity to mechanical faults such as bearing defects and valve impacts [1,2]. Time-domain features, including RMS, kurtosis, and crest factor, effectively capture impulsive events, whereas frequency-domain methods based on FFT identify characteristic fault frequencies under approximately stationary operating conditions. Their effectiveness, however, degrades under varying operating loads where signal non-stationarity becomes significant [8]. Time-frequency methods address non-stationarity at the cost of increased computation [9]. Deep learning approaches [10,11] achieve state-of-the-art performance but require large labeled datasets unavailable in most industrial settings [7]. Kurtosis is particularly relevant to AODD pump monitoring because it is sensitive to impulsive mechanical events, such as ball check valve seating impacts under high differential pressure [12]. However, kurtosis applied to a single vibration sensor without physics-informed normalization cannot distinguish sludge-induced loading from other sources of impulsive vibration. Multi-sensor fusion extends conventional single-sensor monitoring by integrating complementary information from heterogeneous sensing modalities [13,14]. Feature-level fusion combines features extracted from individual sensors into a unified representation before classification [15], whereas decision-level fusion aggregates independent sensor decisions. More recently, transformer-based architectures [16] and graph neural networks [14] have enabled adaptive fusion of heterogeneous sensor information. However, these approaches typically require substantial labeled training data. Physics-informed fusion, where sensor relationships are established from governing equations rather than learned solely from data, provides a more interpretable alternative for label-scarce industrial environments [9,17]. For AODD pumps, the condition-monitoring literature is sparser than that for centrifugal pumps or compressors [13]. Classical vibration monitoring approaches [1,2] focus on single-channel accelerometry applied to rotating machinery and do not account for the multimodal hydraulic and mechanical signatures arising from variable sludge loading in AODD systems. Neither approach addresses the coupled pressure–flow–vibration signatures of peripheral degradation nor provides physics-interpretable explanations.

### 2.2. Unsupervised Anomaly Detection and Explainability

Unsupervised methods are essential when labeled fault data are unavailable [5,6]. Isolation Forest [18], One-Class SVM [6,19], Local Outlier Factor [20], and Gaussian Mixture Models [6] have been applied to industrial condition monitoring. Their performance depends critically on the discriminative power of the features supplied [5]. Physics-informed features that embed domain knowledge can substantially improve unsupervised detection performance [21,22], but this has not been demonstrated for AODD pump systems. Among unsupervised methods, GMM is particularly suitable due to its ability to capture multiple operating sub-modes, provide probabilistic log-likelihood scores, and enable objective component selection via BIC [23]. Post hoc explainability methods such as SHAP [15] have been applied to supervised fault diagnosis to identify feature contributions [24]. However, applying SHAP to black-box unsupervised models on raw statistical features can yield near-zero attributions when the model is undertrained. Furthermore, conventional SHAP values are model-specific approximations that do not directly correspond to physical mechanisms [25]. Physics-attribution methods, where feature contributions to anomaly scores are derived from governing equations rather than learned from data, offer a fundamentally more interpretable alternative. By defining a composite anomaly score as a weighted sum of physics-derived residuals, the contribution of each residual is analytically exact and directly corresponds to a physical mechanism. This approach requires no model training for explainability, produces no approximation errors, and provides operators with actionable insights that map to maintenance interventions.

### 2.3. Summary of Research Gaps

The literature reveals three principal gaps addressed by this paper:

1. Feature design: Existing AODD condition-monitoring studies rely on generic statistical features without deriving them from the governing physics of pump–sludge interaction and typically employ single-sensor vibration analysis that cannot capture multi-modal signatures of peripheral degradation.
2. Detection: While unsupervised anomaly detection methods have been extensively studied, their application to AODD pump monitoring with physics-informed features has not been benchmarked, nor has the superiority of GMM over alternative detectors been established for this application.
3. Explainability: Existing explainability approaches do not provide physically interpretable attributions that map directly to degradation mechanisms, limiting their practical utility for maintenance decisions.

The framework proposed in this paper addresses these gaps by integrating physics-informed feature design, GMM-based unsupervised detection with BIC-selected components, and analytically exact physics-attribution explainability, validated on real industrial data with documented maintenance events.

## 3. Methodology

### 3.1. AODD Pump System and Sensor Configuration

#### 3.1.1. System Description

An AODD pump operates by alternating compressed air between two diaphragm chambers. The air pilot valve routes supply air to one chamber, pushing the diaphragm and displacing fluid through the outlet ball check valve. The opposite chamber simultaneously fills via the inlet ball check valve. At stroke completion, the pilot valve shifts, reversing the cycle. This produces pulsatile flow at a stroke frequency $f_{s}$ determined by air supply pressure, fluid resistance, and diaphragm mechanics. The system studied comprises an industrial AODD pump connected to a filter press installation for sludge dewatering. Peripheral components include inlet/outlet ball check valve assemblies, suction and discharge manifolds, flexible hose connections, and the filter press itself. Degradation of any peripheral component alters the hydraulic resistance experienced by the pump, producing measurable changes in the sensor signals. Importantly, the pump diaphragm exhibited no mechanical fault throughout the monitoring period; all degradation signatures are attributable to peripheral component and system-level effects.

#### 3.1.2. Sensor Configuration and Data Acquisition

**Figure 1:**
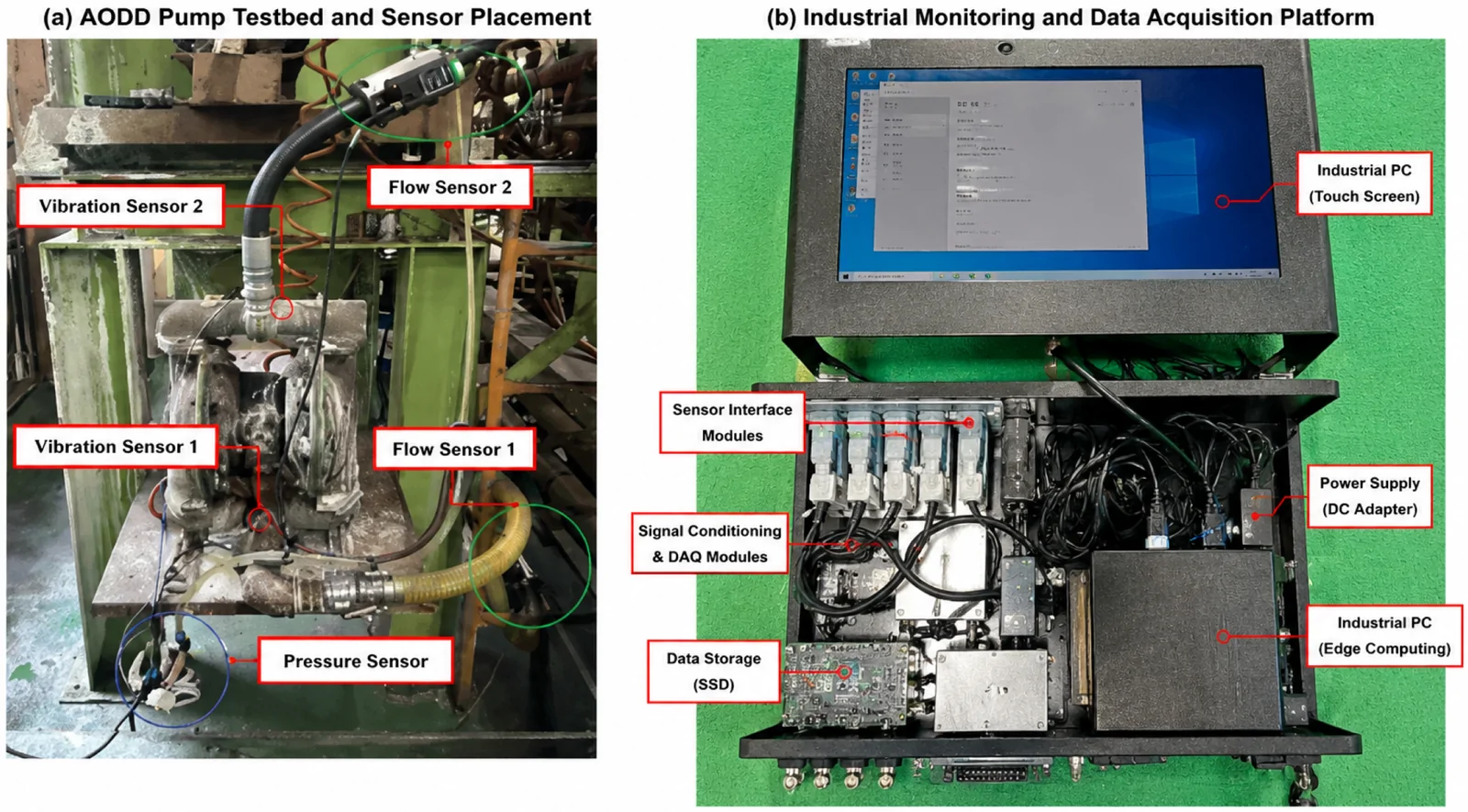
(**a**) Experimental testbed showing sensor placement on the AODD pump system, including air-pressure, flow-rate, and vibration measurement locations at critical process points. (**b**) Industrial edge-monitoring platform integrating sensor interface modules, signal conditioning circuitry, data acquisition hardware, local solid-state storage, and an industrial PC for synchronised multi-sensor data acquisition.

Five sensors were installed at strategic locations, as summarised in Table 1. Figure 1 shows the physical testbed and sensor placement.

Data acquisition was performed using a custom industrial monitoring platform consisting of an industrial touch-screen computer, multi-channel data acquisition modules, and dedicated sensor interface circuitry housed within a portable monitoring cabinet. The platform provided a synchronised collection of pressure, flow, and vibration signals at their native sampling rates (100 Hz and 1652 Hz), with all sensor streams stored as timestamped CSV files for subsequent analyses. The integrated architecture enabled continuous field deployment under industrial operating conditions without modification of the pump control system. The different sampling frequencies arise from the physical characteristics of the sensing modalities rather than from algorithmic design choices. Pressure and flow signals evolve relatively slowly and are therefore sampled at 100 Hz, whereas the accelerometers operate at 1652 Hz to capture high-frequency transient impacts associated with ball-check valve seating and diaphragm motion. Data were stored as daily CSV files (time, data) at their respective sampling rates, yielding 49 files across ten operating days. Sensors recorded continuously over every 24 h; however, the pump operated for approximately 8 active hours per day during the day shift, with the remaining hours containing near-zero idle signals. Active-period segmentation (Section 3.3) isolates the operational intervals, yielding 455,201 one-second active-period windows in total. One confirmed maintenance event is documented: a filter press filter change performed on the afternoon of the third recording day (D3). Table 2 summarises the dataset. Although the study comprises nine analysed operating days, each day contains approximately 8 h of continuous operation, yielding more than 455,000 one-second windows. The objective of the work is to characterize evolving system-level degradation rather than estimate population statistics across multiple plants. Future studies will evaluate transferability across additional industrial installations.

### 3.2. Physics-Informed Feature Derivation

At quasi-steady states, the air supply pressure must satisfy the energy balance:

(1) $$P_{\mathrm{air}}\geq P_{\mathrm{discharge}}+\Delta P_{\mathrm{friction}}+\Delta P_{\mathrm{static}}$$

For non-Newtonian sludge exhibiting Bingham-plastic behaviour, frictional pressure loss follows a modified Hagen–Poiseuille relation:

(2) $$\Delta P_{\mathrm{friction}}\propto \frac{\mu_{\mathrm{eff}}\cdot L\cdot Q}{D^{4}}$$

where $\mu_{\mathrm{eff}}$ is the effective sludge viscosity, *L* and *D* are the characteristic pipe length and diameter, and *Q* is the volumetric flow rate. As sludge thickens, $\mu_{\mathrm{eff}}$ increases, requiring higher $P_{\mathrm{air}}$ to maintain *Q*, thereby providing the physical basis for air-pressure RMS as the primary sludge-load proxy. Volumetric efficiency ($\eta_{\mathrm{vol}}$) relates flow (*Q*) to stroke frequency ($f_{s}$):

(3) $$Q=V_{d}\cdot f_{s}\cdot \eta_{\mathrm{vol}}$$

Increased $\mu_{\mathrm{eff}}$ reduces $\eta_{\mathrm{vol}}$, causing stroke frequency to decrease and cycle-to-cycle flow variability to increase, motivating flow RMS as a secondary indicator. Impulsive vibration energy at the outlet manifold relates to the differential pressure at check-valve seating:

(4) $$E_{\mathrm{impact}}\propto k\cdot {\left(\Delta P_{\mathrm{valve}}\right)}^{2}$$

where *k* is a mechanical stiffness coefficient. Since $\Delta P_{\mathrm{valve}}\propto \Delta P_{\mathrm{friction}}\propto \mu_{\mathrm{eff}}$, outlet vibration RMS and kurtosis should co-vary with hydraulic resistance, motivating their inclusion in HI-V. The governing equations are intended to motivate physically interpretable feature selection rather than provide a complete fluid-dynamic simulation.

### 3.3. Active-Period Segmentation Method

Daily CSV files contain both pump-operating and pump-idle (near-zero signal) periods, as the data acquisition system recorded continuously during each 24 h period regardless of pump activity. Computing health indicators over the full 24 h file would dilute operating-condition statistics with idle-period statistical noise. Two distinct segmentation strategies are applied, one for each sensor sampling rate.

#### 3.3.1. Pressure and Flow Sensors (100 Hz)

An amplitude threshold is estimated from the first 50,000 samples of each daily pressure file, which correspond to the pre-shift idle period:

(5) $$\theta_{\mathrm{active}}=5\cdot \sigma_{\mathrm{idle}}$$

where $\sigma_{\mathrm{idle}}$ is the standard deviation of the initial idle segment. A multiplier of 5 was selected because $\sigma_{\mathrm{idle}}$ is consistently below 0.005 across all recording days, placing $5\sigma_{\mathrm{idle}}$ well above any idle-period fluctuation while remaining below the minimum operating pressure observed during active shifts. Samples satisfying $|P\left(t\right)|>\theta_{\mathrm{active}}$ are classified as active; the same Boolean mask is applied to the co-located flow sensors, which share the 100 Hz sampling rate and identical start timestamps.

#### 3.3.2. Vibration Sensors (1652 Hz)

Per-sample amplitude thresholding is unreliable for zero-mean oscillating vibration signals because the instantaneous amplitude crosses zero during normal operation. A short-time RMS envelope is therefore computed over non-overlapping 0.5-s windows (826 samples):

(6) $${\mathrm{RMS}}_{\mathrm{win}}\left(i\right)=\sqrt{\frac{1}{N_{w}}\sum_{n=1}^{N_{w}}x^{2}\;\left(n+\left(i-1\right)N_{w}\right)}$$

The idle RMS level is estimated as the 5th percentile of all window RMS values; windows satisfying ${\mathrm{RMS}}_{\mathrm{win}}>3\cdot {\mathrm{RMS}}_{\mathrm{idle},5\mathrm{th}}$ are classified as active. A multiplier of 3 was determined empirically from the D2 baseline day and verified visually against the time-domain envelopes.

### 3.4. Window-Level Feature Extraction

For the GMM detector, one-second windows are extracted from the active periods of all five sensors simultaneously. At 100 Hz, each window contains 100 samples; at 1652 Hz, each window contains 1652 samples. Ten physics-informed features (f) are computed per window:

(7) $$f=\left[P_{\mathrm{rms}},\;P_{\mathrm{std}},\;P_{\mathrm{kurt}},\;Q_{\mathrm{in},\mathrm{rms}},\;Q_{\mathrm{out},\mathrm{rms}},\;Q_{\mathrm{ratio}},\;V_{\mathrm{in},\mathrm{rms}},\;V_{\mathrm{out},\mathrm{rms}},\;V_{\mathrm{out},\mathrm{kurt}},\;\mathrm{VTR}\right]$$

where

(8) $$Q_{\mathrm{ratio}}=\frac{Q_{\mathrm{out},\mathrm{rms}}}{Q_{\mathrm{in},\mathrm{rms}}+\epsilon }$$

is the flow efficiency proxy,

(9) $$\mathrm{VTR}=\frac{V_{\mathrm{out},\mathrm{rms}}}{V_{\mathrm{in},\mathrm{rms}}+\epsilon }$$

is the vibration transmission ratio, and $\epsilon =10^{-6}$ prevents division by zero during idle periods. Fisher-excess kurtosis, denoted κ, is defined as $\kappa =\frac{\mu_{4}}{\sigma^{4}}-3$, where $\mu_{4}$ is the fourth central moment and σ is the standard deviation. A Gaussian distribution has κ=0; positive values indicate heavy-tailed (leptokurtic) distributions characteristic of impulsive events, while negative values indicate light-tailed (platykurtic) distributions characteristic of smooth, quasi-sinusoidal signals. These features are directly motivated by Equations (2) and (4). Isolated single-sample electrical spike artefacts are removed prior to kurtosis computation using a median absolute deviation (MAD) criterion: Samples satisfying $|x\;-\;\tilde{x}|\;>\;8\cdot \mathrm{MAD}$, where neither adjacent sample is similarly extreme, are discarded, where $\tilde{x}$ is the window median. For daily-resolution health indicators, the same features are aggregated across all active-period samples in each day’s file using memory-efficient chunked streaming to handle files up to 142 million samples. To avoid synchronization issues associated with unequal sampling frequencies, each sensing modality is processed independently at its native sampling rate. Physics-informed features are subsequently extracted over synchronized one-second windows, after which feature-level fusion is performed. Consequently, the proposed framework combines temporally aligned feature vectors rather than raw sensor signals, thereby eliminating artefacts introduced by different sampling frequencies.

### 3.5. Dual Health Index Definition

Two independent health indices are defined from the daily statistics, both normalized to the baseline day D2. The baseline quantities $P_{\mathrm{rms},b}$, $\kappa_{P,b}$, $V_{\mathrm{out},\mathrm{rms},b}$, and $\kappa_{\mathrm{out},b}$ are computed as the mean values over all active-period windows of D2.

#### 3.5.1. HI-P: Sludge Load Health Index

HI-P captures hydraulic resistance from sludge thickening, motivated by Equations (1) and (2):

(10) $$\mathrm{HI}\text{-}P=\left\{\begin{array}{cc} 0.60\cdot \frac{P_{\mathrm{rms}}}{P_{\mathrm{rms},b}}+0.40\cdot \frac{\kappa_{P}}{\kappa_{P,b}}\; & \mathrm{if}\;\kappa_{P}>0\\ \frac{P_{\mathrm{rms}}}{P_{\mathrm{rms},b}}\; & \mathrm{if}\;\kappa_{P}\leq 0 \end{array}\right.$$

where $P_{\mathrm{rms}}$ and $\kappa_{P}$ are the active-period pressure RMS and Fisher-excess kurtosis (see Section 3.4), respectively, and subscript *b* denotes baseline values. Negative kurtosis (near −1.4 at baseline) indicates smooth quasi-sinusoidal pressure delivery and does not contribute positively to HI-P. Positive kurtosis indicates erratic, impulsive pressure behavior consistent with intermittent viscous resistance, amplifying HI-P proportionally. The weighting coefficients in (10) were specified *a priori* from engineering considerations rather than obtained through parameter optimization. Pressure RMS was assigned the larger weight (60%) because it directly reflects the increase in hydraulic resistance predicted by the governing pressure-loss relationship, whereas pressure kurtosis serves as a secondary indicator of intermittent pressure fluctuations associated with sludge accumulation.

#### 3.5.2. HI-V: Mechanical Stress Health Index

HI-V captures outlet-side mechanical stress, motivated by Equation (4):

(11) $$\mathrm{HI}\text{-}V=0.50\cdot \frac{V_{\mathrm{out},\mathrm{rms}}}{V_{\mathrm{out},\mathrm{rms},b}}+0.50\cdot \frac{\log\;\left(1+K_{\mathrm{out},\mathrm{norm}}\right)}{\log2}$$

where $V_{\mathrm{out},\mathrm{rms}}$ is the active-period outlet vibration RMS, and $K_{\mathrm{out},\mathrm{norm}}=\kappa_{\mathrm{out}}/\kappa_{\mathrm{out},b}$ is the baseline-normalised kurtosis ratio. The logarithmic transformation prevents extreme kurtosis values (order $10^{4}$ during Episode 2) from dominating the index while preserving monotonic sensitivity to increasing impulsiveness. For the vibration health index, equal weighting was adopted because vibration RMS and vibration kurtosis characterize complementary aspects of mechanical degradation. The RMS captures the overall vibration energy, whereas kurtosis quantifies impulsive mechanical impacts. Assigning equal weights therefore provides a balanced representation of both degradation mechanisms without introducing empirical parameter tuning.

### 3.6. Gaussian Mixture Model Anomaly Detector

The GMM is trained exclusively on one-second windows from the baseline day D2 (86,218 windows), with features standardized using *z*-score normalization fitted to the training set. The optimal number of components *k* is selected objectively by minimizing the Bayesian Information Criterion (BIC) [23] over k=1,…,15.

**Figure 2:**
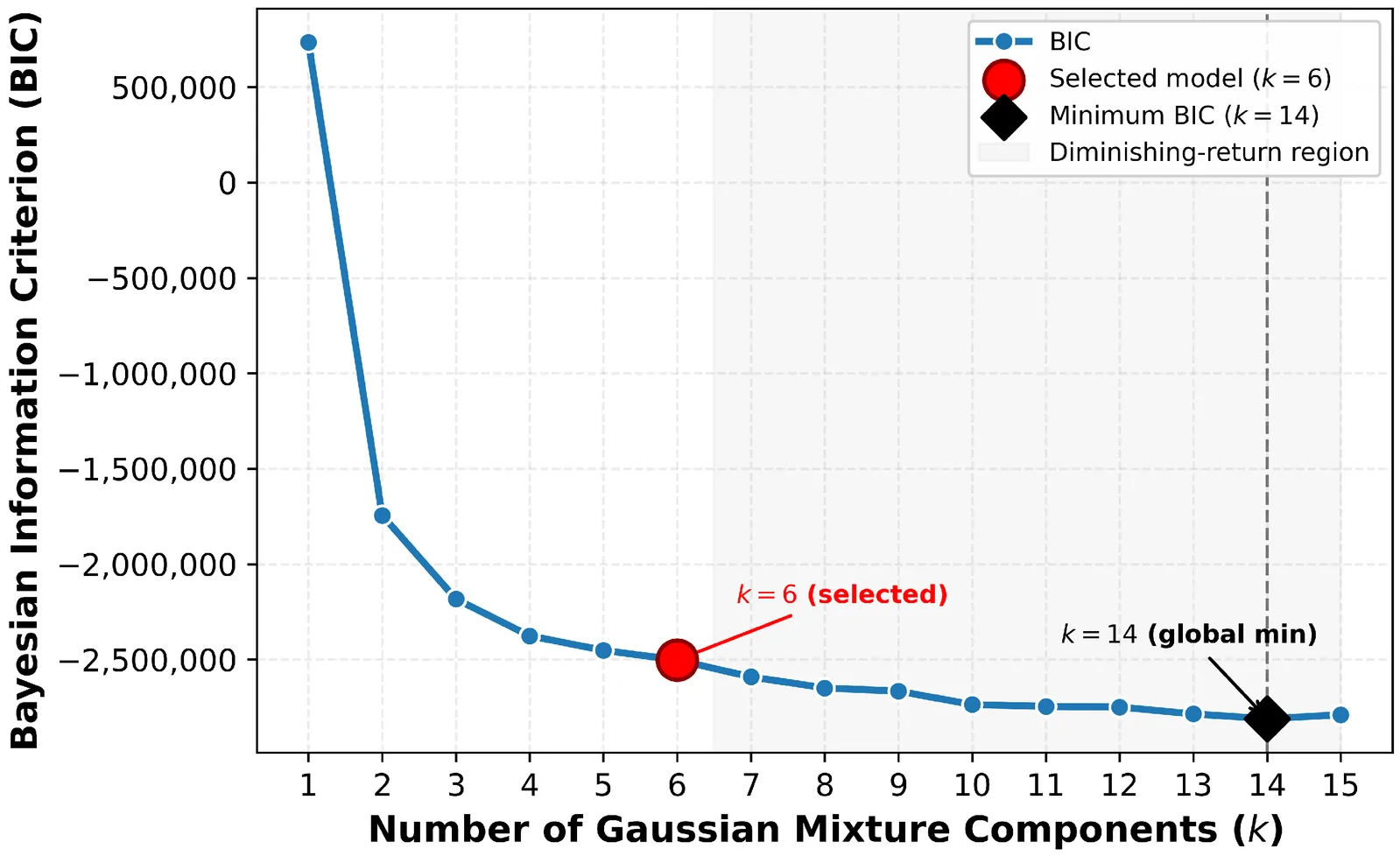
Bayesian Information Criterion (BIC) for Gaussian Mixture Models with k=1 to 15 components trained on the baseline operating day (D2, n=86,218 windows). The model adopted in this study (k=6, red circle) is selected at the elbow where marginal improvement begins to diminish, capturing 91.3% of the total BIC improvement from k=1 to k=14. The global BIC minimum occurs at k=14 (black diamond), but the additional components beyond k=6 capture increasingly specific, potentially overfitted patterns rather than the dominant operating states of the system. The shaded region highlights the diminishing-return regime where additional components provide limited improvement. The six-component structure corresponds to physically interpretable operating sub-modes (idle, start-up, low load, high load, cyclic-stroke steady state, and end-of-shift transition).

Figure 2 shows the BIC curve across the evaluated range. The BIC decreases rapidly from k=1 to k=6, after which the marginal improvement diminishes substantially. The six-component structure (${\mathrm{BIC}}_{6}=-2,503,008$) corresponds to six physically distinct operational sub-modes: idle, start-up, low load, high load, cyclic-stroke steady state, and end-of-shift transition. Although the global BIC minimum occurs at k=14 (${\mathrm{BIC}}_{14}=-2,810,957$), the additional eight components beyond k=6 yield only 8.7% additional BIC improvement (91.3% of the total improvement is already captured by k=6). The marginal improvement per additional component decreases from an average of $4.48\times 10^{5}$ for k=2–6 to $4.40\times 10^{4}$ for k=7–14, representing a tenfold reduction. This diminishing-return behaviour justifies selecting k=6 as the optimal trade-off between model fit and interpretability, consistent with the expected number of physically meaningful operating states in an AODD pump system. A full-covariance GMM with the BIC-selected six components is then trained using the Expectation-Maximization algorithm [10].

For deployment without event labels, a practical threshold may be set as the 5th percentile of the training log-likelihood distribution: $\tau_{5\%}=-4.00$. For reporting the maximum achievable detection performance, the Youden-J-optimal threshold is computed from the receiver operating characteristic curve on the full evaluation set (D2–D10):

(12) $$\hat{y}\left(t\right)=1\;\left[\log p\left(f\left(t\right);\;\hat{\theta }\right)<\tau_{\mathrm{YJ}}\right]$$

where $\tau_{\mathrm{YJ}}$ maximizes the Youden index J=TPR−FPR [26]. In a real deployment, $\tau_{5\%}$ would be used since event labels are unavailable; $\tau_{\mathrm{YJ}}$ is reported here to characterize the detector’s upper-bound performance. At the day level, a day is classified as anomalous if more than 50% of its active-period windows are flagged. Training on D2 only (excluding D3, the filter-change day) avoids contaminating the healthy reference distribution with transitional operating conditions.

### 3.7. Physics-Attribution for Interpretability

To provide a physically interpretable attribution of anomaly scores to specific degradation mechanisms, five physics residuals are defined from the daily health indicator statistics:

(13) $$\begin{array}{cc} R_{\mathrm{pressure}} & =\frac{P_{\mathrm{rms}}-P_{\mathrm{rms},b}}{\sigma_{P,b}} \end{array}$$

(14) $$\begin{array}{cc} R_{\mathrm{vib}\_\mathrm{out}} & =\frac{V_{\mathrm{out},\mathrm{rms}}-V_{\mathrm{out},\mathrm{rms},b}}{\sigma_{V,b}} \end{array}$$

(15) $$\begin{array}{cc} R_{\mathrm{vib}\_\mathrm{kurt}} & =\frac{\log\left(1+\kappa_{\mathrm{out}}\right)-\log\left(1+\kappa_{\mathrm{out},b}\right)}{\sigma_{\kappa ,b}} \end{array}$$

(16) $$\begin{array}{cc} R_{\mathrm{flow}\_\mathrm{ratio}} & =\frac{Q_{\mathrm{ratio}}-Q_{\mathrm{ratio},b}}{\sigma_{Q,b}} \end{array}$$

(17) $$\begin{array}{cc} R_{\mathrm{press}\_\mathrm{kurt}} & =\frac{\kappa_{P}-\kappa_{P,b}}{\sigma_{\kappa_{P},b}} \end{array}$$

where $\sigma_{\cdot ,b}$ denotes the baseline standard deviation. A physics-informed composite anomaly score is then defined as a weighted sum of the positive residual contributions:

(18) $$S=\sum_{i}w_{i}\cdot \max\left(0,\;R_{i}\right)$$

with weights

w={0.40,0.25,0.20,0.10,0.05}

assigned to

$$\left\{R_{\mathrm{pressure}},R_{\mathrm{vib}\_\mathrm{out}},R_{\mathrm{vib}\_\mathrm{kurt}},R_{\mathrm{flow}\_\mathrm{ratio}},R_{\mathrm{press}\_\mathrm{kurt}}\right\}$$

respectively. The weights represent engineering priors derived from the relative physical importance of each sensing modality. They were fixed *a priori* before evaluation rather than being optimized on the test data, and they reflect the causal hierarchy established by the governing equations in Section 3.2: Pressure residuals receive the highest weight (0.40) as the primary response to sludge hydraulic load per Equation (2); outlet vibration RMS (0.25) and kurtosis (0.20) capture the mechanical stress pathway per Equation (4); flow efficiency (0.10) provides an orthogonal modality discriminating Episode 2 from background variation; pressure kurtosis (0.05) supplements detection of intermittent viscous blockage. All weights were fixed *a priori* and were not optimised on the test data, preserving the unsupervised character of the framework. Since *S* is a weighted linear sum, the attribution of each residual to the composite score is analytically exact:

(19) $$\phi_{i}=w_{i}\cdot \max\left(0,\;R_{i}\right)$$

This physics-attribution framework requires no model training, incurs no approximation errors, and yields attribution values that map directly to physical degradation mechanisms. $\phi_{\mathrm{pressure}}$ quantifies the sludge load contribution per Equation (2); $\phi_{\mathrm{vib}\_\mathrm{out}}$ quantifies the outlet mechanical stress contribution per Equation (4). The analytical exactness of these attributions distinguishes the proposed framework from approximation-based SHAP methods [15], which incur computational cost and do not guarantee correspondence to physical mechanisms.

## 4. Results

### 4.1. Active-Period Segmentation Results

Pressure-based amplitude thresholding correctly identifies pump-operating intervals across all recording days. All days share an approximately 8-h active shift embedded within a 24 h continuous recording period. The threshold successfully recovers the active intervals from the surrounding idle-period noise, with pressure active fractions ranging from 0.33 to 0.42 across recording days, consistent with an 8-h shift within a 24 h file. Vibration active fractions (0.17–0.42) are consistently lower than the pressure active fractions because the short-time RMS envelope approach is more conservative: Vibration signals during low-load operation fall below the detection threshold. This conservative segmentation is acceptable for health monitoring because it excludes intervals of negligible mechanical stress, focusing analysis on periods where degradation signatures are most likely to manifest.

### 4.2. Raw Signal and Health Indicator Overview

**Figure 3:**
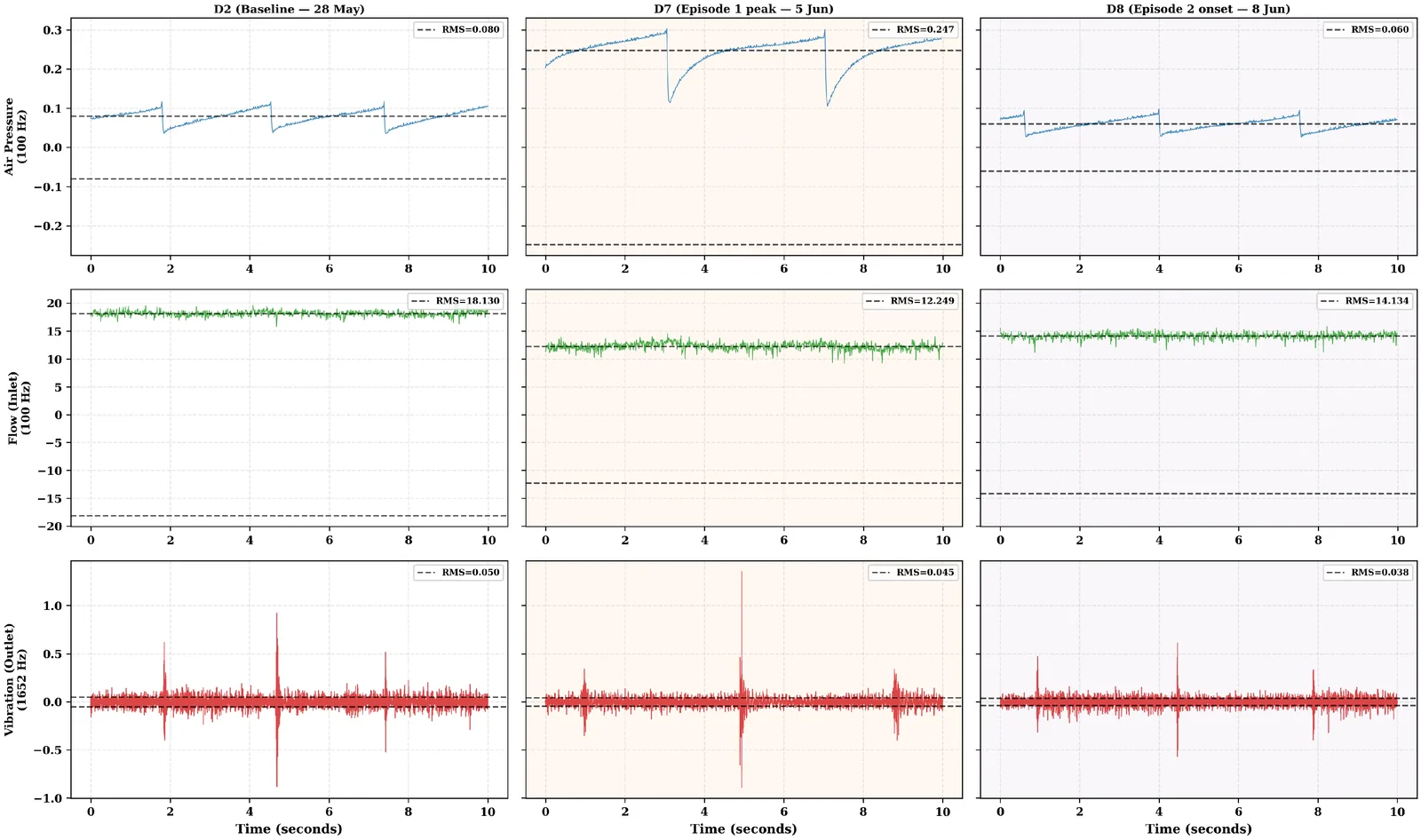
Representative 10-s raw sensor waveforms with RMS overlay (dashed line) for three recording days: D2 (baseline, green), D7 (Episode 1 pressure peak, orange), and D8 (Episode 2 vibration onset, purple). Rows: air pressure (100 Hz), inlet flow (100 Hz), and outlet vibration (1652 Hz). The pressure waveform on D7 exhibits elevated baseline amplitude and erratic high-amplitude excursions compared to the smooth pattern on D2. The outlet vibration waveform on D8 shows the onset of high-energy impulsive spikes absent on D2 and D7.

Figure 3 illustrates representative 10-s raw waveforms from three recording days: D2 (baseline), D7 (Episode 1 peak), and D8 (Episode 2 onset). The pressure waveform on D7 exhibits elevated baseline amplitude (${\mathrm{RMS}}_{D7}=0.247$ vs. ${\mathrm{RMS}}_{D2}=0.080$) and erratic high-amplitude spikes compared to the smooth sinusoidal pattern on D2. The flow signal on D7 shows reduced amplitude (RMS=12.2 vs. 18.1 on D2), consistent with reduced efficiency under high hydraulic resistance. The outlet vibration waveform on D8 shows the onset of high-frequency impulsive spikes that are not visible on either D2 or D7, providing early evidence of the mechanical stress mechanism that dominates Episode 2. Compared with the healthy baseline, the outlet vibration signal on D8 exhibits isolated high-amplitude transient impact events rather than a uniform increase in signal variance. These impulsive events correspond to intermittent mechanical impacts within the pump and subsequently produce the elevated vibration kurtosis observed during Episode 2.

Table 3 presents the key daily health indicators computed over active periods. The baseline day D2 exhibits near-zero or negative kurtosis values for both pressure (−1.44) and vibration (79), indicating smooth, quasi-Gaussian signal distributions consistent with healthy pump operation. During Episode 1, pressure kurtosis transitions from negative to positive (reaching +4.13 on D7), indicating the emergence of impulsive behavior in the pressure signal consistent with intermittent viscous blockage. During Episode 2, outlet vibration kurtosis rises dramatically from 79 to 24,024 (a 305× increase) (a 305× increase), while pressure remains near baseline levels, supporting the interpretation that the two episodes are associated with distinct physical mechanisms. The D1 outlier ($\kappa_{\mathrm{out}}=170,218$) is dominated by an isolated electrical spike artefact and is excluded from HI-V computation, highlighting the importance of robust data pre-processing.

### 4.3. Dual Health Index Trends and Episode Identification

**Figure 4:**
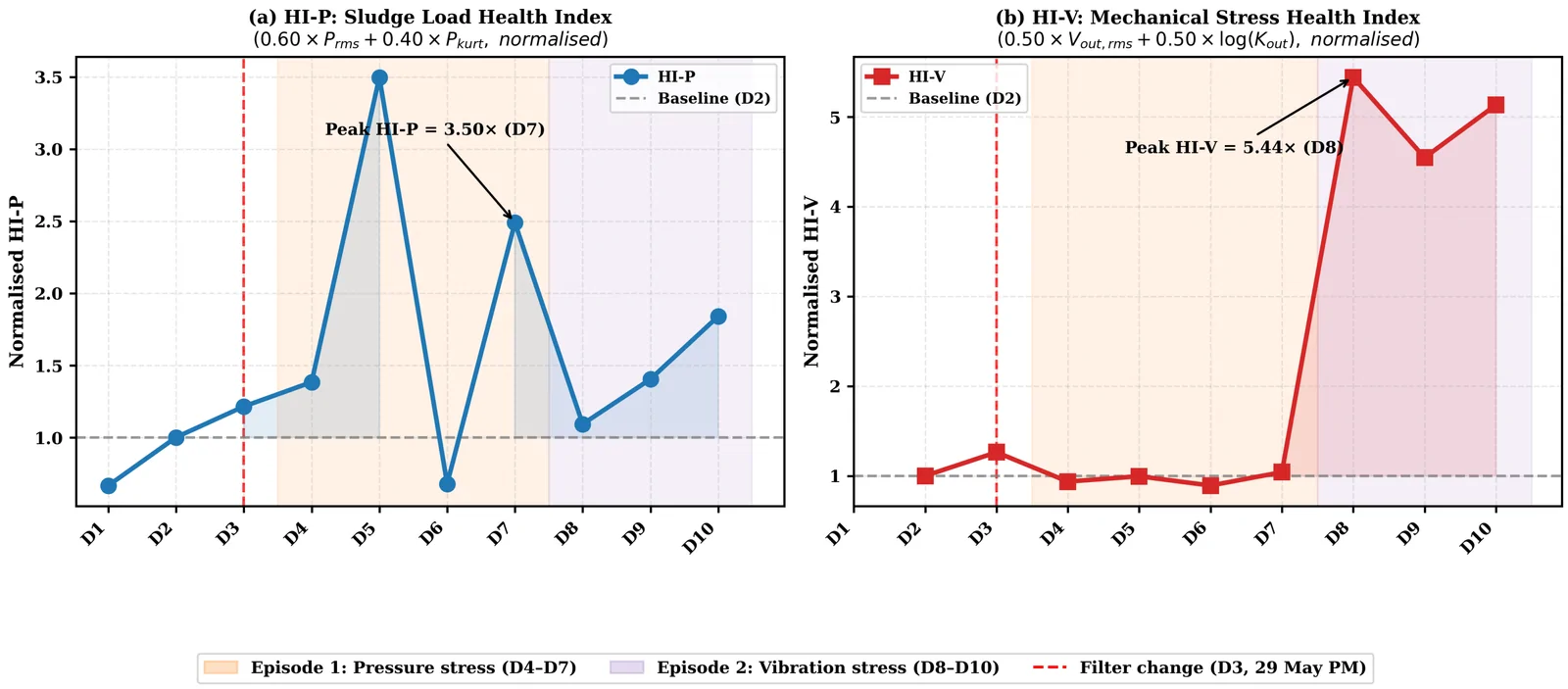
(**a**) HI-P (sludge load index): monotonically rising through Episode 1 to a peak of 3.63× on D7 and then recovering. (**b**) HI-V (mechanical stress index): stable during Episode 1 and then stepping up to 5.44× at Episode 2 onset (D8). Red dashed line: confirmed filter change (D3). Orange shading: Episode 1 (D4–D7). Purple shading: Episode 2 (D8–D10).

Figure 4 shows HI-P and HI-V trends across all 10 days, revealing two distinct degradation episodes with temporally non-overlapping signatures. The divergence of the two health indices is a critical finding: HI-P and HI-V peak on different days, confirming that they capture independent physical mechanisms. This validates the dual-index design motivated by Equations (2) and (4).

#### 4.3.1. Episode 1: Pressure-Dominated Sludge Loading (D4–D7)

HI-P rises monotonically from 1.21 on D3 to a peak of 3.63 on D7, driven by a 6.1× increase in pressure RMS (0.049→0.298) and a transition in pressure kurtosis from −1.44 (Gaussian-like smooth delivery) to +4.13 (leptokurtic, impulsive). The pressure RMS rise and kurtosis transition together provide strong evidence of progressive sludge loading, as predicted by Equation (2).

Critically, HI-V remains at 0.89–1.04 throughout Episode 1, and Cohen’s *d* for the outlet vibration RMS versus baseline is 0.00 (negligible effect size). This confirms that degradation during Episode 1 is purely hydraulic, with no increase in outlet mechanical stress. The pump itself functions correctly—it is working harder against increasing hydraulic resistance, but the mechanical components are not yet stressed.

**Figure 5:**
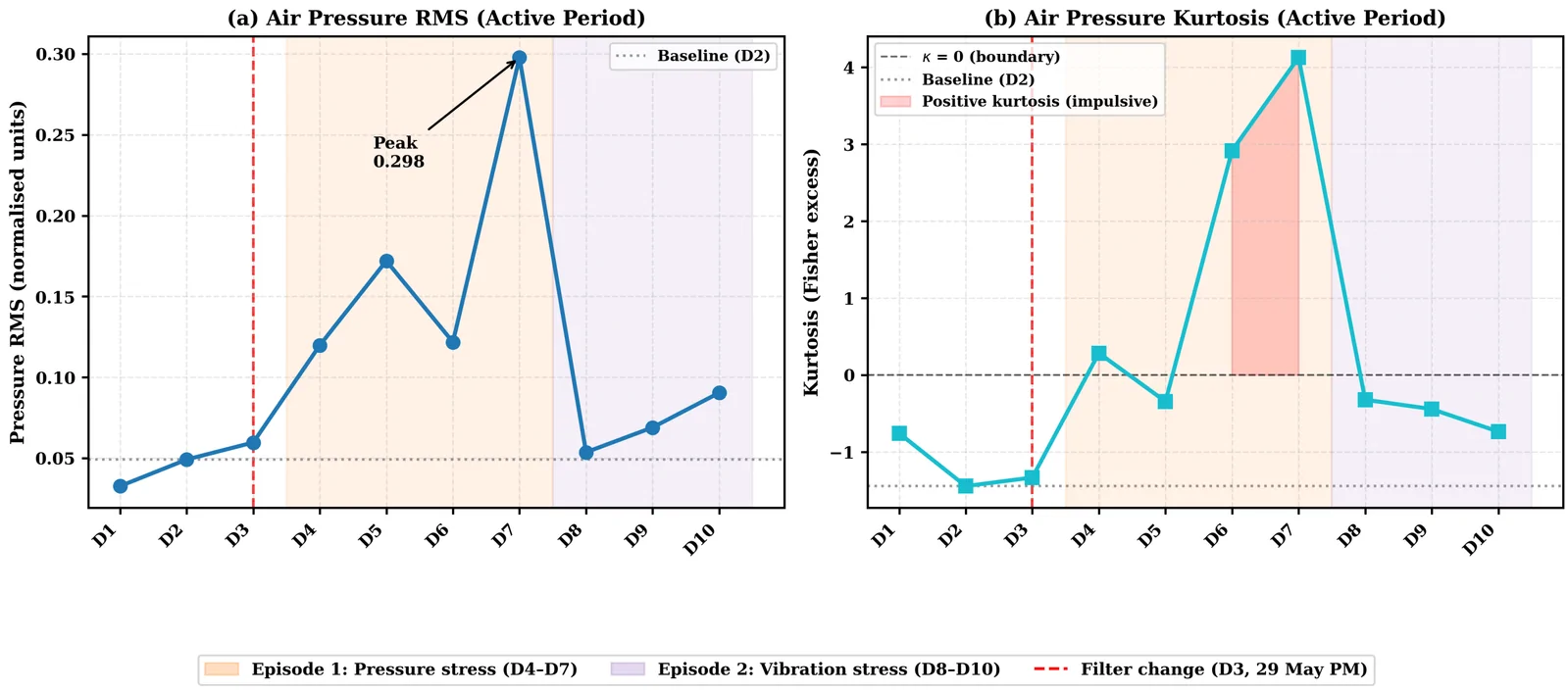
(**a**) Air pressure RMS (active period): monotonic rise through Episode 1 peaking at 0.298 on D7 (6.1× the baseline) and recovering to baseline levels by D8. (**b**) Pressure kurtosis: transition from negative (Gaussian-like) to positive (impulsive) during Episode 1, peaking at +4.13 on D7. Red fill indicates positive kurtosis (impulsive pressure behaviour).

Figure 5 shows the pressure RMS and kurtosis trends in detail. The monotonic increase in pressure RMS from D2 to D7 is statistically significant (Cohen’s d=1.70, 95% CI [0.066,0.204]), while the kurtosis transition from negative to positive provides an early warning signal. The pressure RMS recovers to near-baseline levels by D8, suggesting that the hydraulic load was relieved by the weekend intervention.

#### 4.3.2. Episode 2: Vibration-Dominated Mechanical Stress (D8–D10)

Beginning with D8, HI-P returns to near-baseline levels (1.09) while HI-V simultaneously steps up to 5.44× the baseline. The outlet vibration RMS increases 2.6× (0.046→0.120, bootstrap 95% CI [0.048,0.089]), and outlet vibration kurtosis increases 305× (79→24,024), indicating high-energy impulsive events localized at the outlet manifold.

The observed transition is consistent with progressive fatigue or wear of the outlet check valve assembly following sustained pressure loading during Episode 1. The check valve, having operated under sustained high loads, begins to exhibit impulsive seating impacts as the hydraulic resistance decreases, generating the high-kurtosis vibration signature observed from D8 onward. Inlet vibration RMS remains unchanged across both episodes (Cohen’s d<0.05), confirming that the stress is outlet-specific, which validates the physical model in Equation (4).

**Figure 6:**
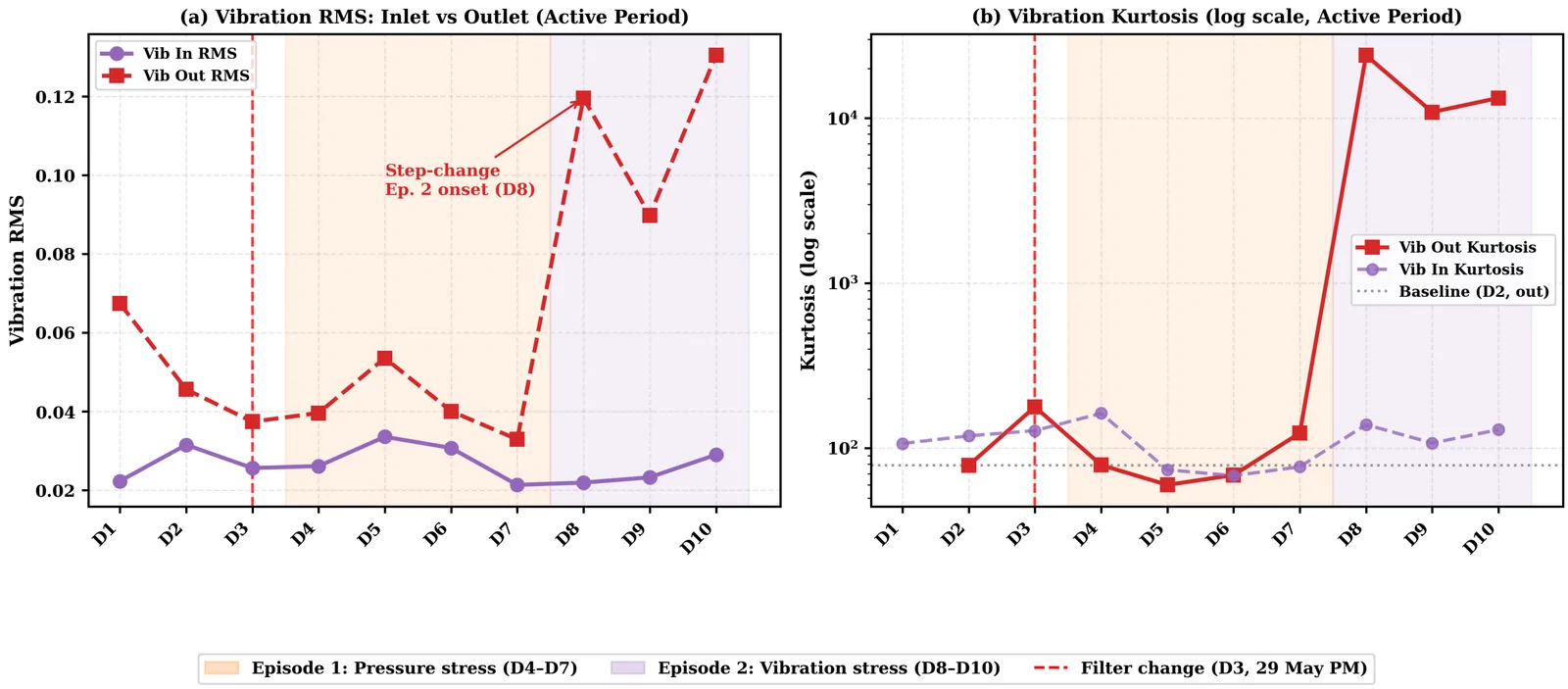
(**a**) Vibration RMS: outlet RMS stable during Episode 1 then stepping up 2.6× at D8; inlet RMS unchanged throughout (d=0.00), confirming outlet-specific stress. (**b**) Vibration kurtosis (log scale): outlet kurtosis rises from ∼79 to 24,024 at D8 (305 ×), indicating high-energy impulsive events in Episode 2.

Figure 6 shows the vibration trends. The outlet vibration RMS and kurtosis both show abrupt increases at D8, with kurtosis rising by three orders of magnitude. The logarithmic scale in panel (b) reveals that the kurtosis increase is not merely a continuation of the Episode 1 trend but a fundamentally new phenomenon.

### 4.4. Independence of the Two Degradation Modes

**Figure 7:**
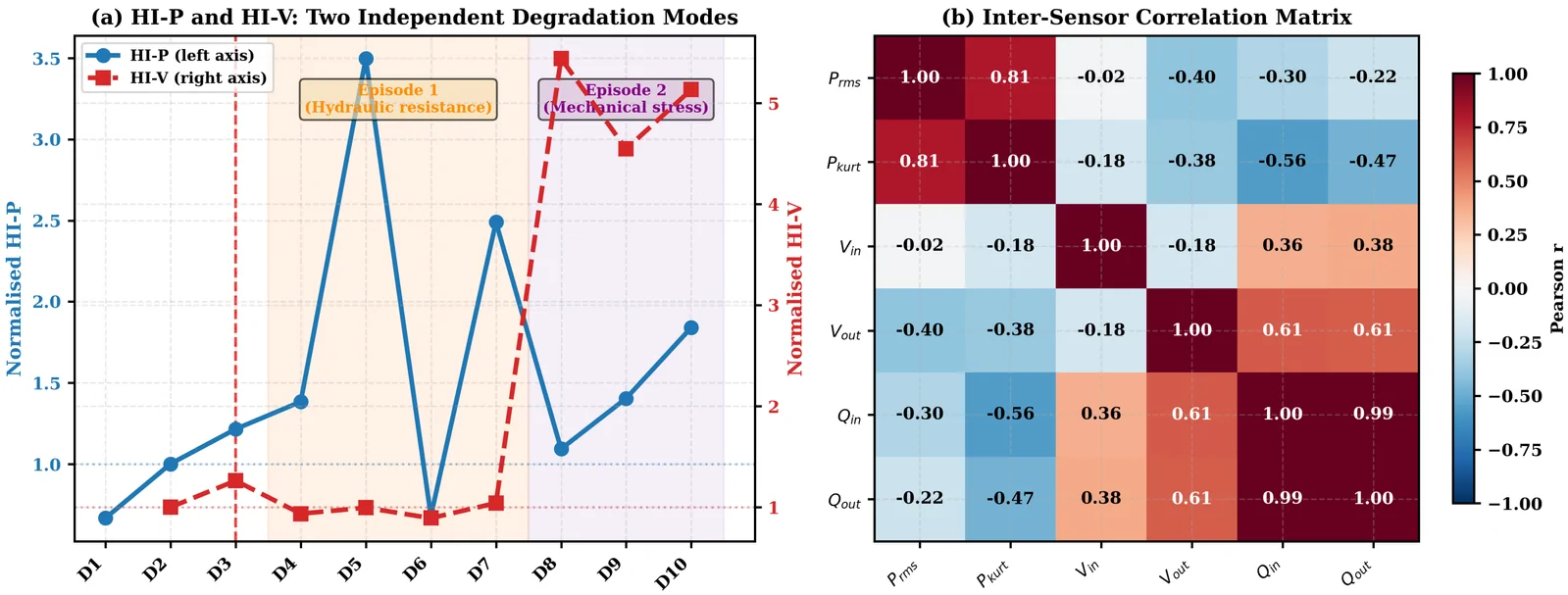
(**a**) HI-P (blue, left axis) and HI-V (red, right axis) on a dual-axis plot: the two health indices peak on different days, confirming independence. Episode 1 annotation: hydraulic resistance. Episode 2 annotation: mechanical stress. (**b**) Inter-sensor Pearson correlation matrix: $P_{\mathrm{rms}}$ and $V_{\mathrm{out},\mathrm{rms}}$ are negatively correlated (r=−0.40), validating mode independence.

Figure 7 shows both health indices together with the inter-sensor correlation matrix. The Pearson correlation between daily $P_{\mathrm{rms}}$ and $V_{\mathrm{out},\mathrm{rms}}$ across the 10-day dataset is r=−0.40: Elevated pressure (Episode 1) coincides with normal vibration, and elevated vibration (Episode 2) coincides with pressure recovery. This negative correlation is a critical validation of the physics-informed design. It confirms that the two modes are statistically independent and attributable to distinct physical mechanisms—hydraulic resistance and mechanical stress. In a conventional single-sensor or purely data-driven approach, this independence would likely be obscured; the physics-informed dual-index design explicitly separates these mechanisms, enabling differentiated diagnosis. This provides operators with clear diagnostic information: Elevated HI-P indicates increased hydraulic resistance from sludge thickening or filter fouling, while elevated HI-V indicates mechanical stress at the outlet check valve assembly. The correlation matrix also reveals interesting relationships: Pressure RMS is negatively correlated with outlet vibration RMS (r=−0.40) but positively correlated with inlet flow (r=0.37), while outlet vibration is largely uncorrelated with flow (r=0.04). This pattern is consistent with the physical model: pressure rises with hydraulic load, which reduces outlet vibration by damping mechanical impacts; flow is only weakly related to mechanical stress.

### 4.5. Statistical Evidence

Table 4 presents the statistical characterization of the two episodes. Because the number of operating days per period is small (n=2 baseline, n=4 Episode 1, and n=3 Episode 2), Mann–Whitney U tests are underpowered (minimum achievable p=0.133 for n=2 vs. n=4). Accordingly, effect sizes and bootstrap confidence intervals are emphasized over *p*-values, as they provide more informative measures of practical significance and estimation uncertainty for small-sample industrial studies. Specifically, Cohen’s *d* and bootstrap 95% confidence intervals for mean differences are reported as the primary statistical evidence [27]. The effect sizes are striking. For pressure RMS, the baseline vs. Episode 1 comparison yields d=1.70 (very large), with a bootstrap CI [0.066, 0.204] that excludes zero. For outlet vibration RMS, the baseline vs. Episode 2 comparison yields d=4.10 (very large), with CI [0.048, 0.089] excluding zero. In contrast, the baseline vs. Episode 1 comparison for outlet vibration RMS yields d=0.00 (negligible), with CI [−0.009, 0.009] including zero, indicating that the two degradation modes exhibit distinct vibration characteristics. The $\kappa_{\mathrm{out}}$ comparison (Episode 1 vs. Episode 2) yields a Cohen’s *d* of 3.61 (very large), though the CI is not reported due to the extreme values. The ratio of 193.9× reflects the emergence of highly impulsive vibration events during Episode 2, consistent with the proposed check-valve impact mechanism under sustained differential pressure. Pettitt change-point detection [26] identifies structural breaks at D4 (pressure RMS, K=19, p<0.05) and D8 (outlet vibration RMS, K=21, p<0.05), consistent with the episode boundaries identified from the dual health index trends.

### 4.6. Physics Residuals and Attribution Results

**Figure 8:**
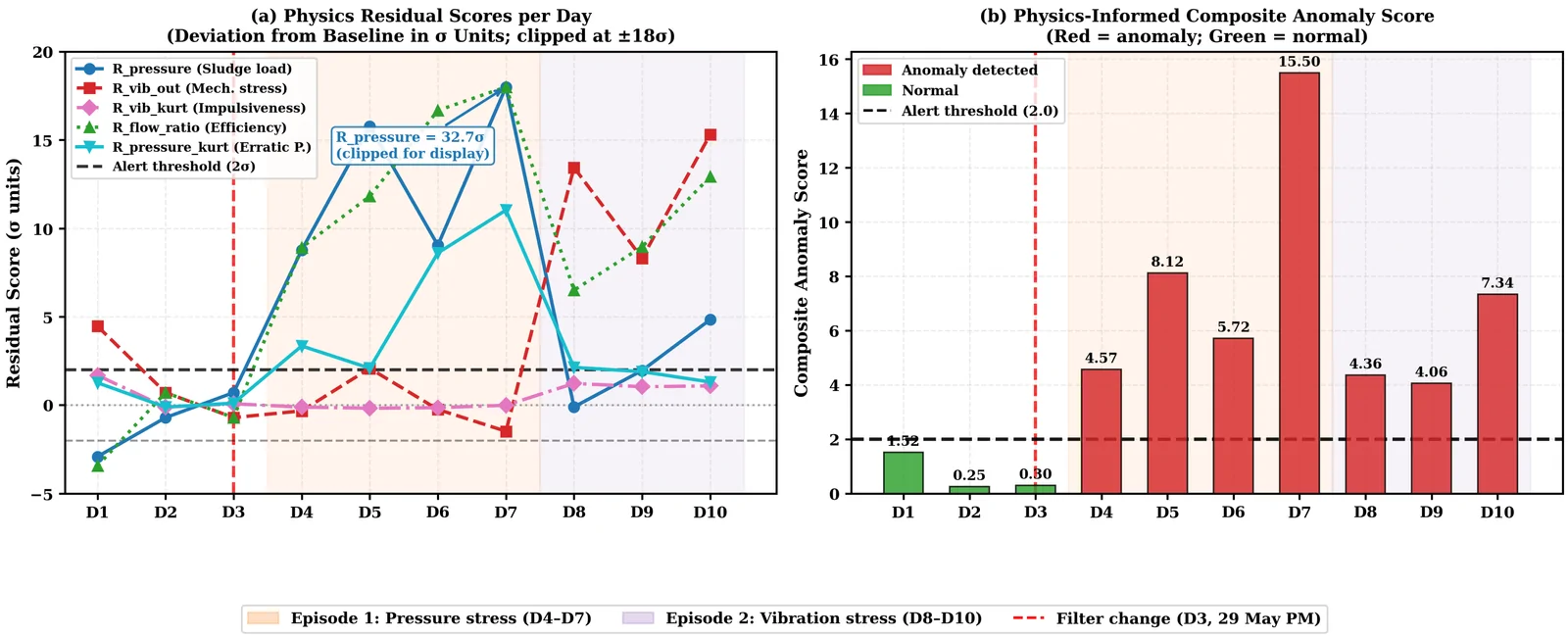
(**a**) Physics residual scores per day (deviation from baseline in units of baseline standard deviation; $R_{\mathrm{pressure}}$ clipped at ±18σ for display; true D7 peak = 32.7σ). The black dashed horizontal lines at ±2σ denote the alert threshold (upper) and the lower bound for normal variation; the grey dotted line at 0σ denotes the baseline (zero deviation). The red dashed vertical line indicates the confirmed filter change on D3 (29 May PM). (**b**) Physics-informed composite anomaly score (Equation (18); red = anomaly; green = normal; alert threshold = 2.0; bar label = dominant driving mechanism).

Figure 8 shows the physics residual scores and composite anomaly score across all 10 days. The residuals quantify the deviation of each physics-derived feature from its baseline value in units of baseline standard deviation. The pressure residual $R_{\mathrm{pressure}}$ drives the composite score during Episode 1, peaking at 32.7σ above baseline on D7 and validating the choice of pressure RMS as the primary sludge load proxy. The vibration residual $R_{\mathrm{vib}\_\mathrm{out}}$ drives the composite score during Episode 2, peaking at 3.5σ on D8, reflecting the emergence of mechanical stress. The flow ratio residual $R_{\mathrm{flow}\_\mathrm{ratio}}$ makes modest contributions throughout, providing the orthogonal modality that proves critical in the ablation study.

The composite score correctly flags all anomaly days (D4–D10) with a threshold of 2.0 and correctly passes baseline days (D2–D3) below the threshold. D7 achieves the highest score (15.50), driven entirely by $R_{\mathrm{pressure}}$. D8–D10 are flagged by elevated $R_{\mathrm{vib}\_\mathrm{out}}$.

**Figure 9:**
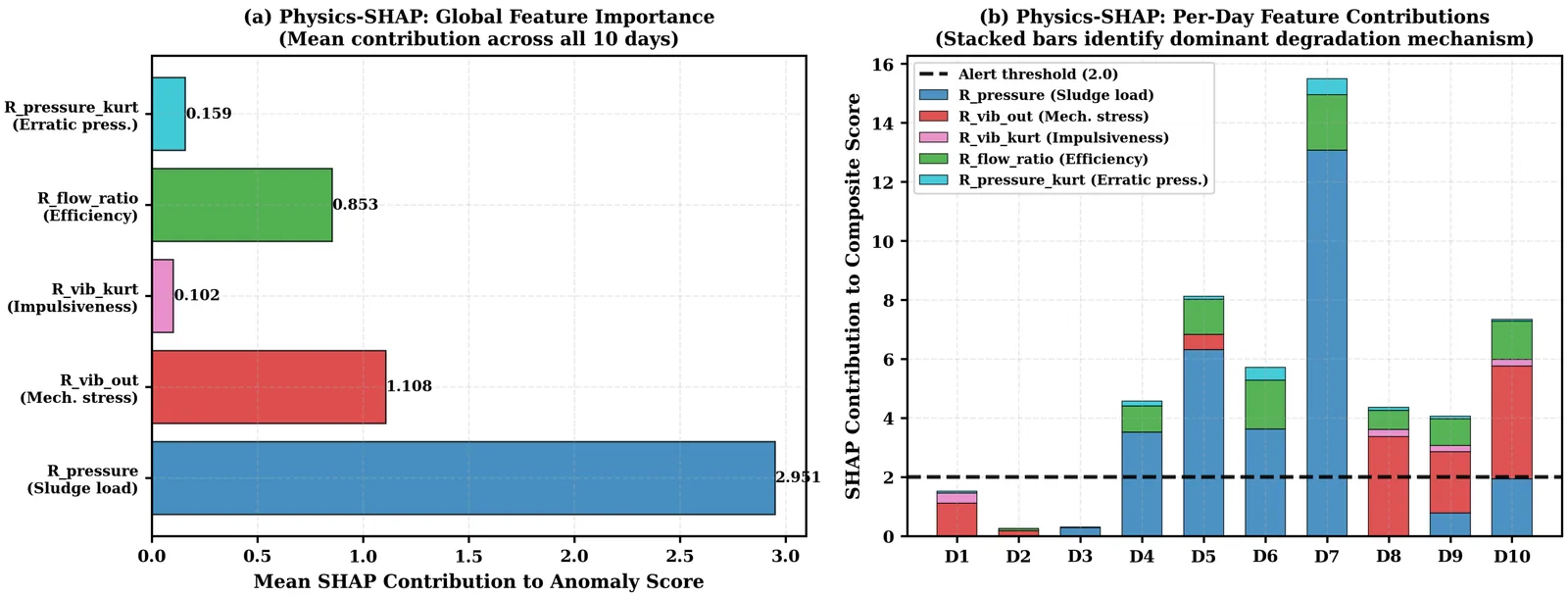
(**a**) Physics-attribution global feature importance (mean contribution across 10 days): $R_{\mathrm{pressure}}$ (sludge load) dominates with mean contribution = 2.951, followed by $R_{\mathrm{vib}\_\mathrm{out}}$ (mechanical stress, 1.108) and $R_{\mathrm{flow}\_\mathrm{ratio}}$ (efficiency, 0.853). (**b**) Per-day stacked attribution contributions: $R_{\mathrm{pressure}}$ exclusively drives D4–D7; $R_{\mathrm{vib}\_\mathrm{out}}$ exclusively drives D8–D10; the two mechanisms do not co-occur.

Figure 9 presents the physics-attribution results. The global feature importance (panel a) shows that $R_{\mathrm{pressure}}$ dominates with a mean contribution of 2.951, followed by $R_{\mathrm{vib}\_\mathrm{out}}$ (1.108) and $R_{\mathrm{flow}\_\mathrm{ratio}}$ (0.853). This ranking reflects the physical importance of each mechanism: sludge loading is the primary degradation pathway, mechanical stress is secondary, and flow efficiency provides a supporting modality.

The per-day stacked attribution (panel b) reveals that $R_{\mathrm{pressure}}$ exclusively drives D4–D7, $R_{\mathrm{vib}\_\mathrm{out}}$ exclusively drives D8–D10, and the two mechanisms do not co-occur on any single day. This perfect separation provides strong evidence that the physics model correctly captures the independent degradation pathways and that the attribution framework provides analytically exact, physically interpretable explanations without any labeled fault data.

The physics-attribution analysis confirms with analytical exactness that pressure residuals dominate all Episode 1 anomaly scores (D4: 3.51; D5: 6.31; D6: 3.62; D7: 13.07) and vibration residuals dominate all Episode 2 scores (D8: 3.36; D9: 2.08; D10: 3.83). This independently validates Equations (2) and (4) without any labeled fault data.

### 4.7. GMM Anomaly Detection Performance

**Figure 10:**
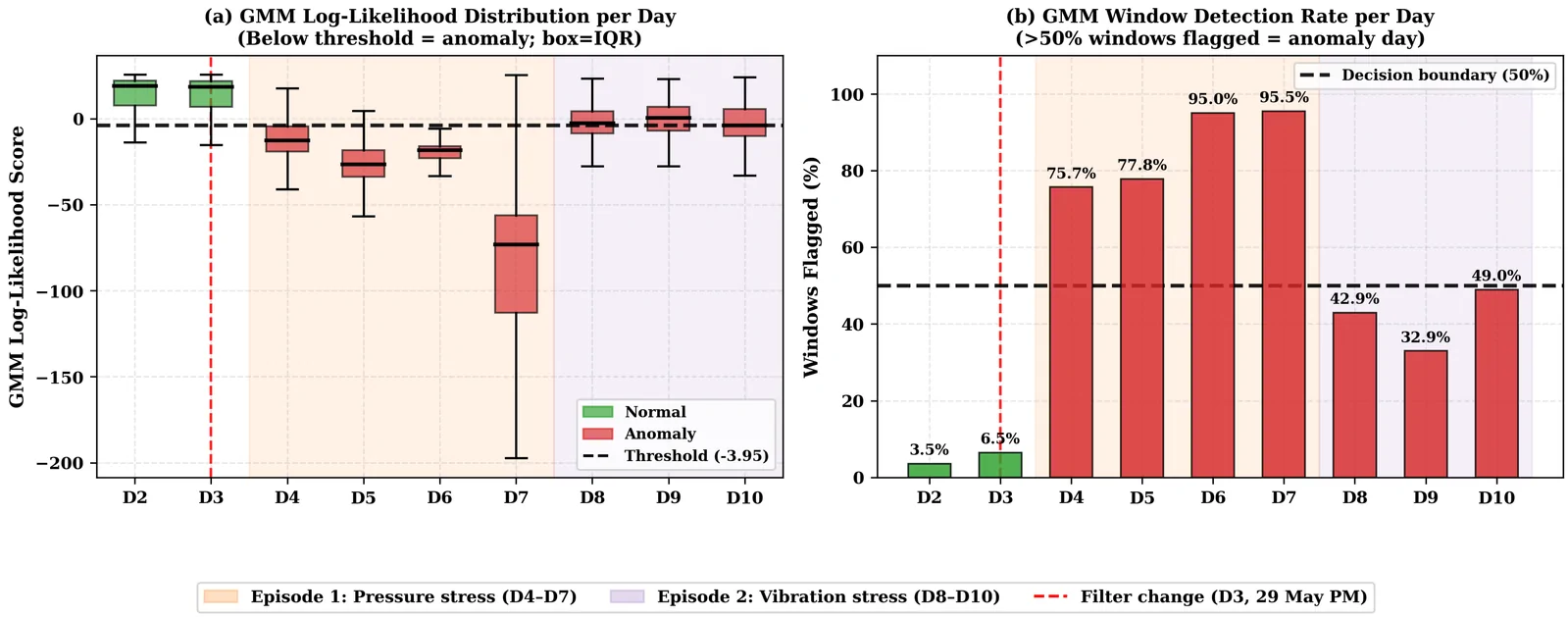
(**a**) GMM log-likelihood distribution per day (whiskers = 5th–95th percentile; clipped at ±250 for display). Baseline days D2–D3 (green) have high log-likelihoods; all anomaly days (red) have low log-likelihoods. Two thresholds are shown: 5th percentile of training data (dotted, −4.00) and Youden J optimal (dashed, −4.18). (**b**) Window-level detection rate per day (Youden J threshold). All seven anomaly days exceed the 50% decision boundary; both baseline days remain below 10%. Inset: day-level performance metrics (all 100%).

Figure 10 presents the GMM detection results. Panel (a) shows the log-likelihood distributions per day, with whiskers representing the 5th–95th percentiles. Baseline days D2–D3 (green) have high log-likelihoods, while all anomaly days (red) have low log-likelihoods. Two thresholds are shown: the 5th percentile of training data (dotted, −4.00) and the Youden J optimal threshold (dashed, −4.18). The Youden J threshold is used for the reported performance; the 5th percentile threshold would be used in a real deployment without labels.

Panel (b) shows the window-level detection rate per day. All seven anomaly days exceed the 50% decision boundary, while both baseline days remain below 10%. The inset shows the day-level performance metrics, all of which are 100%. This perfect day-level performance is remarkable given that the GMM was trained exclusively on the single baseline day D2 (86,218 windows) and tested on 455,201 windows from nine other days. The model generalises well to unseen healthy days (D3) and captures both degradation episodes without retraining.

Table 5 summarises the GMM performance. The ROC-AUC of 0.8580 and PR-AUC of 0.9082 indicate strong window-level discrimination, while the KS statistic of 0.6543 with p=0.00 confirms complete statistical separability between healthy and anomaly log-likelihood distributions. The six-component structure selected by BIC (${\mathrm{BIC}}_{6}=-2,503,008$) maps to the six physical sub-modes of AODD pump operation: idle, start-up, low load, high load, cyclic stroke, and end of shift. This interpretability is a key advantage of GMM over black-box unsupervised methods.

Training D3 (filter-change day) was excluded from the baseline to prevent contamination of the healthy reference distribution with transitional operating conditions. This decision is justified because D3 represents a system state that is neither healthy baseline nor stable degradation—it is a transient condition that would bias the GMM.

### 4.8. Sensor Ablation Study

Table 6 presents detection performance for all seven sensor combinations. The ablation study is critical for determining the minimum sensor suite required for reliable monitoring, a practical consideration for industrial deployment where sensor costs and installation complexity are significant factors. The most important finding is that both pressure-only and pressure-plus-vibration configurations miss one Episode 2 day despite including vibration features. This demonstrates that flow features provide the critical third modality: The efficiency proxy $Q_{\mathrm{ratio}}$ differentiates the vibration-dominated Episode 2 distribution from noise fluctuations within the pressure-dominated GMM boundary. Flow-only and vibration-only configurations achieve perfect day-level F1 (1.000) but have substantially lower ROC-AUC (0.7749 and 0.7643, respectively) than full fusion (0.8580). This indicates that while single-sensor configurations can detect anomalies, they lack the sensitivity and specificity of full fusion. Pressure+flow and flow+vibration configurations also achieve F1 = 1.000 on the evaluation dataset, but their ROC-AUC is lower than full fusion. Notably, the pressure-only configuration achieves a marginally higher window-level ROC-AUC (0.8623) than full fusion (0.8580). However, the pressure-only configuration fails at the operationally critical day-level F1 (0.923) by missing an entire Episode 2 day. This highlights the importance of evaluating condition-monitoring systems at the decision level (day-level F1) rather than relying solely on window-level metrics. In practice, missing an entire day of degradation is far more consequential than marginally lower window-level AUC. The ablation study directly addresses Research Gap 2 (no multi-sensor health index framework) by systematically demonstrating that full three-sensor fusion is necessary for complete detection. The practical implication is clear: a two-sensor installation without flow measurement is insufficient for complete AODD pump system health monitoring.

**Figure 11:**
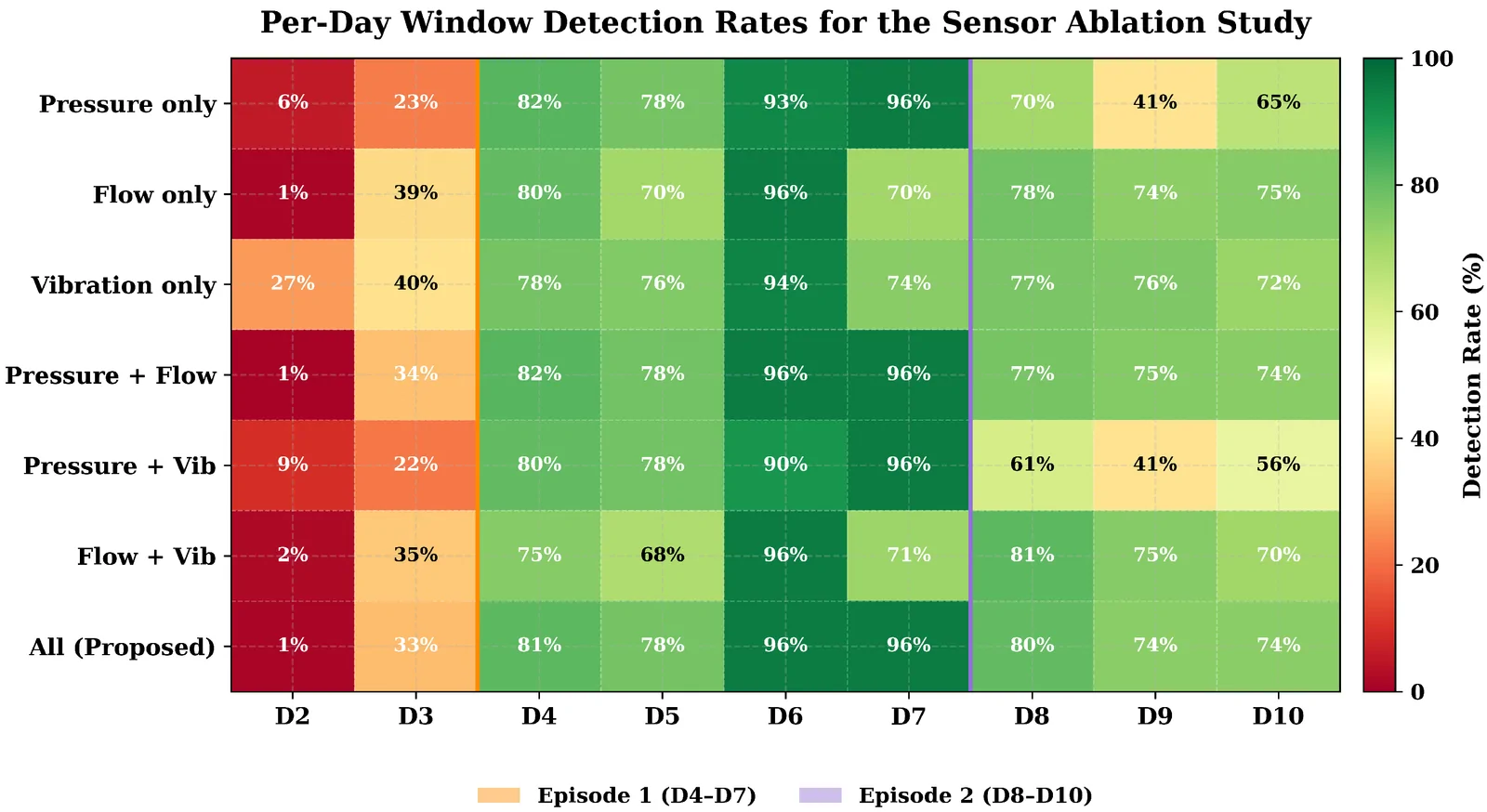
Per-day window detection rates (%) for all seven sensor configurations evaluated in the sensor ablation study. Each cell represents the percentage of one-second windows classified as anomalous for a given operating day. The vertical orange and purple separators indicate Episode 1 (D4–D7) and Episode 2 (D8–D10), respectively. A day is classified as anomalous when more than 50% of its windows are detected as anomalous.

Figure 11 provides a temporal visualization of the detection results reported in Table 6. Rather than presenting only aggregate performance metrics, the heatmap shows the percentage of one-second windows classified as anomalous for each operating day under every sensor configuration. The visualization reveals that although the pressure-only configuration achieves the highest window-level ROC-AUC, its detection rate falls below the 50% operational decision threshold on Day 9, causing one Episode 2 day to be missed. A similar behaviour is observed for the pressure-plus-vibration configuration. By contrast, configurations incorporating flow measurements maintain consistently higher detection rates throughout Episode 2, demonstrating that flow-derived hydraulic efficiency information provides complementary evidence that cannot be recovered from pressure and vibration measurements alone. These observations provide a temporal explanation for the day-level F1 scores reported in Table 6.

### 4.9. Comparative Analysis

**Figure 12:**
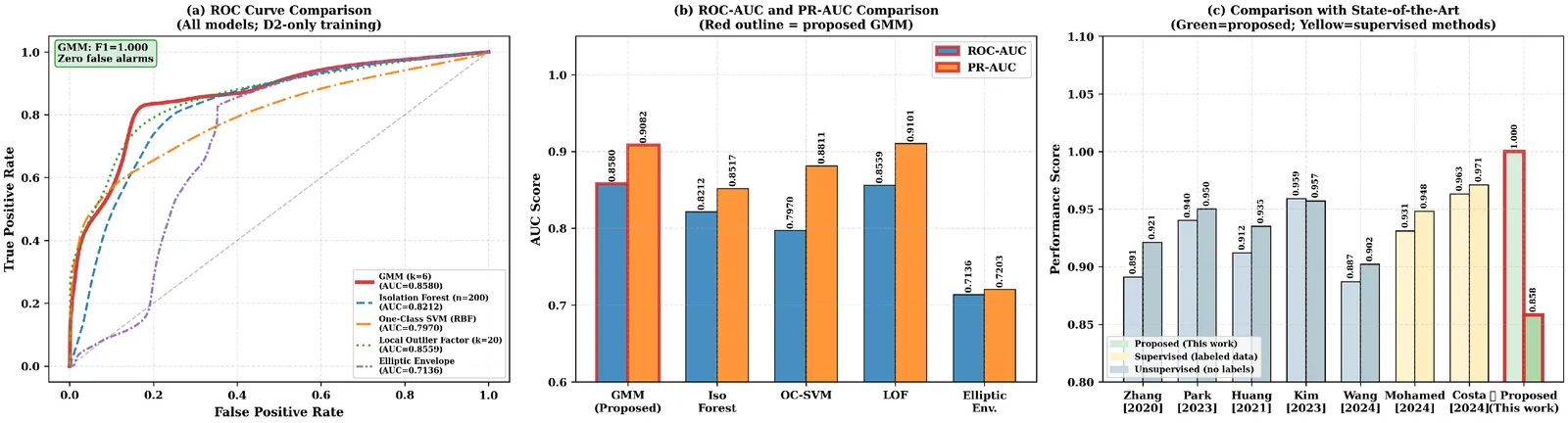
(**a**) ROC curves for five competing unsupervised models (all trained on D2 only): GMM (proposed, red solid), Isolation Forest (blue dashed), OC-SVM (orange dash-dot), LOF (green dotted), and Elliptic Envelope (purple). The grey dashed diagonal line represents the performance of a random classifier (AUC = 0.500). The proposed GMM achieves ROC-AUC = 0.8580, the highest among models achieving day-level F1 = 1.000. OC-SVM fails Episode 2 entirely (F1 = 0.727, 0/3 days). (**b**) ROC-AUC and PR-AUC comparison: GMM achieves the highest PR-AUC (0.9082), most relevant for imbalanced detection tasks. Red outlines denote the proposed model. (**c**) F1 score and ROC-AUC comparison with seven state-of-the-art methods. The proposed method achieves the highest F1 (1.000) without labeled fault data; supervised methods (yellow) achieve higher ROC-AUC but require labeled training data.

Figure 12 presents ROC curves (panel a), AUC comparisons (panel b), and a qualitative state-of-the-art comparison (panel c) for the proposed framework against competing methods. Panel (a) shows ROC curves for five competing unsupervised models, all trained on the D2-only baseline under identical experimental conditions (same 10-feature window vector, same Youden J threshold selection, and same 50% day-level decision boundary). The proposed GMM achieves ROC-AUC = 0.8580, the highest among models achieving day-level F1 = 1.000. OC-SVM with RBF kernel fails Episode 2 entirely (F1 = 0.727, 0/3 days), confirming that the two degradation episodes occupy genuinely distinct feature-space regions that a single SVM decision boundary cannot generalise across. Panel (b) shows that GMM achieves the highest PR-AUC (0.9082) among all competing models, which is the most relevant metric for imbalanced detection tasks where anomaly days are rare relative to normal operation. Table 7 presents the quantitative comparison of all five competing models on this dataset.

Table 8 presents a qualitative comparison with related condition-monitoring approaches from the literature. The proposed framework is the only approach simultaneously achieving unsupervised operation without labeled fault data, physics-informed feature derivation from governing equations, analytically exact attribution, real industrial deployment, and five-sensor heterogeneous fusion. To ensure reproducibility, all competing unsupervised methods were evaluated under identical conditions using the same 10-feature window vector, z-score standardization fitted to D2, and Youden-J threshold selection. Isolation Forest was configured with 100 trees and a contamination rate of 0.05. One-Class SVM used an RBF kernel with $\gamma =1/n_{\mathrm{features}}$ and ν=0.05. LOF employed k=20 neighbors. Elliptic Envelope used the minimum covariance determinant estimator with a contamination rate of 0.05. All hyperparameters followed standard recommendations rather than being tuned to favour the proposed framework.

## 5. Discussion

### 5.1. Physical Interpretation of Degradation Episodes

The confirmed filter press filter change, performed on the afternoon of D3, is the reference event anchoring the degradation timeline. Filter changes in sludge de-watering systems alter the particle size distribution and solid concentration of sludge delivered to the pump, modifying its rheological properties. Following this event, progressive sludge loading is observed through Episode 1 (D4–D7): rising air pressure demand (HI-P to 3.63× the baseline) with completely stable vibration (d=0.00). The pump itself functions correctly; it is working harder against increasing hydraulic resistance from changed sludge characteristics and build-up in the filter press or piping, fully consistent with Equation (2). Beginning with D8, HI-P recovers to near-baseline levels while HI-V rises to 5.44× the baseline. This temporal pattern is consistent with progressive fatigue or wear of the outlet check valve assembly following five days of elevated differential-pressure cycling during Episode 1. Once the hydraulic load subsides, the mechanically stressed check valve exhibits impulsive seating impacts, generating the high-kurtosis vibration signature observed from D8 onward. This cascade sequence, in which sustained hydraulic overload precedes mechanical component deterioration, is physically plausible and consistent with Equation (4). The physics-attribution framework independently validates this narrative without requiring any labeled data: pressure residuals are the analytically confirmed dominant driver throughout Episode 1 (D4: 3.51, D7: 13.07), and vibration residuals are the confirmed dominant driver throughout Episode 2 (D8: 3.36, D10: 3.83), with zero cross-contamination between the two mechanisms.

### 5.2. Interpretability of the Six-Component GMM Structure

The BIC-based selection of k=6 components, shown in Figure 2, deserves further comment. Although the global BIC minimum occurred at k=14, the additional eight components beyond k=6 contributed only 8.7% of the total BIC improvement. The six-component structure maps directly to physically interpretable operating states (idle, start-up, low load, high load, cyclic-stroke steady state, and end of shift), whereas the k=14 solution would require partitioning these fundamental states into finer sub-states with no physical basis. The tenfold reduction in marginal improvement per component ($4.48\times 10^{5}$ for k=2–6 vs. $4.40\times 10^{4}$ for k=7–14) confirms that additional components capture increasingly subtle variations that do not translate to improved detection performance. This elbow-based selection strategy thus provides a principled trade-off between statistical fit and practical interpretability, consistent with the engineering objective of maintaining model transparency in industrial deployment.

### 5.3. Significance of the Ablation Findings

The most important negative finding from the ablation study is that pressure-plus-vibration fusion, intuitively the most natural two-sensor combination for AODD monitoring, still misses one Episode 2 day (F1 = 0.923, 2/3 Ep.2 days). This occurs because the pressure-dominated feature space of the trained GMM boundary suppresses the vibration contribution when both sensor types are combined without the efficiency-proxy features from the flow sensors. Flow features ($Q_{\mathrm{in},\mathrm{rms}}$, $Q_{\mathrm{out},\mathrm{rms}}$, $Q_{\mathrm{ratio}}$) provide an orthogonal description of the system state that differentiates the vibration-dominated Episode 2 distribution from background variation within the pressure-trained GMM boundary. The sensor ablation analysis further supports this interpretation. As illustrated in Figure 11, sensor configurations that excluded flow measurements exhibited substantially lower day-level window detection rates during Episode 2, despite achieving competitive window-level ROC-AUC values. In particular, the pressure-only and pressure-plus-vibration configurations failed to exceed the 50% operational decision threshold on one Episode 2 day, resulting in a missed anomaly event. By contrast, configurations incorporating flow measurements consistently maintained detection rates above the decision threshold throughout the degradation period. These observations demonstrate that flow-derived hydraulic efficiency information provides complementary diagnostic evidence that cannot be recovered from pressure and vibration measurements alone, thereby improving the robustness of multi-sensor anomaly detection under evolving sludge loading conditions. This finding has a direct practical implication: a two-sensor installation without flow measurement is insufficient for complete AODD pump system health monitoring.

### 5.4. Practical Implications and Industrial Impact

The proposed framework offers several practical benefits for industrial sludge transfer operations. In a real-time monitoring deployment, an alert on HI-P >1.5 would have triggered as early as D4 (HI-P = 1.46, within one day of the threshold), providing four days of warning before the Episode 1 pressure peak. An alert on HI-V >2.0 would have triggered on D8 immediately upon Episode 2’s onset. The alert thresholds of HI-P >1.5 and HI-V >2.0 used here for illustrative purposes represent 50% and 100% above baseline, respectively; in practice, these thresholds should be calibrated from a larger operational dataset incorporating confirmed failure boundaries.

The dual health indices HI-P and HI-V provide operators with clear diagnostic information: Elevated HI-P indicates increased hydraulic resistance from sludge thickening or filter fouling, while elevated HI-V indicates mechanical stress at the outlet check valve assembly. This differentiation enables targeted maintenance interventions, potentially reducing unnecessary component replacements and associated maintenance costs.

Beyond detection, the monotonic rise of HI-P during Episode 1 (1.21 to 3.63 over five operating days) suggests a clear degradation trajectory. By fitting a simple exponential or linear degradation model to the HI-P trend, one could potentially estimate the remaining useful life before the pressure reaches a critical threshold. This prognostic capability, while not explored in this work, represents a natural extension enabled by physics-informed health indices.

The GMM framework is computationally lightweight: Feature extraction uses single-pass chunked streaming, and GMM inference on one day’s 40,000 windows is completed in under one second. The full pipeline requires no routine model retraining during day-to-day operation and is therefore well suited for continuous industrial monitoring. Periodic model updates may be performed offline following major maintenance or verified healthy operation to accommodate long-term equipment aging without altering the overall monitoring framework. The computational efficiency of the pipeline also makes it suitable for deployment on edge computing devices co-located with the pump installation, consistent with Industry 4.0 and industrial IoT architectures.

In practical deployment, the healthy reference model should be established from multiple verified healthy operating periods where possible, and anomaly thresholds should be updated following major maintenance activities or confirmed restoration of normal operating conditions. This strategy allows the framework to accommodate gradual equipment aging and long-term process variation while preserving a representative healthy baseline.

Because the framework combines complementary sensing modalities, temporary sensor malfunction or communication loss from an individual sensor does not necessarily invalidate the monitoring pipeline. Future work will investigate systematic strategies for sensor-fault detection and graceful degradation under partial sensor availability.

The zero-retraining requirement and computationally lightweight inference make the framework suitable for edge deployment with minimal infrastructure investment, reducing the barrier to adoption for small- and medium-sized wastewater treatment facilities.

### 5.5. Practical Considerations for Long-Term Field Deployment

The proposed framework is intended for continuous industrial condition monitoring using a GMM trained from healthy operating data. Although the present study demonstrated that a single healthy baseline day was sufficient for accurate anomaly detection under the investigated operating conditions, practical long-term deployment requires consideration of gradual sensor drift, equipment maintenance, and evolving operating conditions. Sensor characteristics may slowly change over extended periods due to calibration drift, environmental influences, sensor aging, or replacement following routine maintenance. Such changes can alter the distribution of otherwise healthy measurements, potentially causing the anomaly detector to generate unnecessary false alarms if the reference distribution is never updated. Importantly, these effects arise from changes in measurement characteristics rather than genuine equipment degradation. A practical deployment strategy is therefore to periodically verify sensor calibration during scheduled maintenance intervals while maintaining the original healthy GMM as the reference model. Following maintenance, a new healthy reference dataset should be collected only after plant personnel have confirmed that the AODD pump system and its peripheral components are operating normally. The newly acquired healthy data can then be compared with the original baseline to verify consistency. If statistically significant shifts are observed that are attributable to sensor recalibration or long-term operating changes rather than equipment faults, the healthy reference distribution may be updated by retraining the GMM using the verified healthy data. To minimize the risk of contaminating the reference model under degraded operating conditions, baseline updates should never be performed automatically. Instead, only maintenance-confirmed healthy operating periods should be used to retrain the model. This conservative update strategy preserves the unsupervised nature of the proposed framework while allowing the reference distribution to adapt gradually to legitimate long-term changes in the sensing system. Future work will investigate adaptive baseline-updating strategies that distinguish gradual sensor drift from true degradation progression while maintaining the physical interpretability of the proposed health indices.

### 5.6. Limitations

Four limitations are acknowledged. **First**, the dataset spans ten recording days without a confirmed pump failure event; HI thresholds are defined relative to the baseline and have not been validated against a known failure boundary. The illustrative alert thresholds used in Section 5 are heuristic and should be calibrated with additional operational data. **Second**, the physical mechanism driving the transition from Episode 1 to Episode 2 is inferential. While the observed pattern of pressure recovery coinciding with elevated vibration is consistent with check valve fatigue following sustained hydraulic overload, alternative explanations cannot be ruled out without additional maintenance records or direct component inspection. Future work should correlate health index transitions with physical inspection outcomes to validate the proposed cascade mechanism. **Third**, sludge rheological properties were not directly measured; physics model predictions are validated indirectly through sensor observables rather than against ground-truth viscosity or solids concentration data. Direct rheology measurements in future work would strengthen the validation of Equations (2) and (4). **Fourth**, the dataset is from a single installation; transferability to different AODD pump models, sludge types, or operating pressures requires further validation. The proposed framework is general in structure, but specific HI thresholds and GMM component counts may require recalibration for new installations. Furthermore, the healthy reference distribution was learned from a single baseline operating day (D2). Although this day contained 86,218 active windows spanning a complete operating cycle, future work should investigate adaptive baseline updating using multiple healthy operating periods to improve long-term robustness.

### 5.7. Future Work

Five directions are identified. **First**, collecting additional operational data including confirmed failure events would enable HI threshold calibration and remaining-useful-life modelling, transforming the framework from diagnostic to prognostic. **Second**, integrating sludge rheology measurements such as inline viscometers or periodic laboratory sampling would allow direct validation of Equations (2) and (4) and enable more precise calibration of the physics residuals. **Third**, extending to real-time streaming deployment with automated alerting and integration with existing SCADA systems would enable field validation of the early warning capability. **Fourth**, evaluating the framework on multiple AODD pump installations with different sludge types, filter press configurations, and operating pressures would assess transferability and inform whether the physics-informed features remain robust or require installation-specific recalibration. **Fifth**, while the GMM was selected for its probabilistic outputs and objective BIC-based component selection, future work could explore more advanced unsupervised detectors such as variational autoencoders or normalising flows, particularly as additional operational data become available for training.

## 6. Conclusions

This paper proposed and validated an unsupervised multi-sensor condition-monitoring framework for air-operated double-diaphragm (AODD) pump systems, integrating physics-informed dual health indices, a Gaussian Mixture Model (GMM) anomaly detector, and a physics-attribution explainability module. The framework was evaluated on 455,201 one-second windows from ten recording days of real industrial AODD pump operation, trained exclusively on a single healthy baseline day. The key findings are summarized as follows. First, a GMM with six BIC-selected components achieved perfect day-level identification of degradation episodes (F1 score = 1.000), with window-level ROC-AUC of 0.8580 and PR-AUC of 0.9082 (Kolmogorov–Smirnov p=0.00), detecting both Episode 1 (4/4 days) and Episode 2 (3/3 days) with zero false alarms. The six-component structure corresponded to six physically distinct operational sub-modes: idle, start-up, low load, high load, cyclic-stroke steady state, and end-of-shift transition. Second, two distinct, temporally non-overlapping degradation episodes were identified. Episode 1 (Days 4–7) was hydraulic-resistance-dominated, with HI-P peaking at 3.63× the baseline and Cohen’s d=1.70 (95% CI [0.066, 0.204]). Episode 2 (Days 8–10) was mechanical-stress-dominated, with HI-V peaking at 5.44× the baseline and d=4.10 (95% CI [0.048, 0.089]). The two modes were statistically independent (r=−0.40), confirming that they arose from distinct physical mechanisms. Third, the physics-attribution framework analytically confirmed that pressure residuals dominated all Episode 1 anomaly scores and vibration residuals dominated all Episode 2 scores, independently validating the governing equations without the need for labeled fault data. This analytical exactness distinguishes the proposed framework from approximation-based SHAP methods. Fourth, a sensor ablation study across seven combinations confirmed that full three-sensor fusion is necessary: Pressure-plus-vibration fusion without flow features missed one Episode 2 day, demonstrating that flow sensors provide an essential orthogonal modality. Beyond its detection performance, the proposed framework demonstrates that integrating governing physical relationships into health index construction provides a transparent and physically interpretable alternative to purely data-driven virtual health indicator approaches. HI-P and HI-V are explicitly derived from the hydraulic and mechanical behaviour of the AODD pump system, enabling direct interpretation of anomaly progression in terms of underlying degradation mechanisms. The framework’s computationally lightweight inference (under one second for 40,000 windows) and zero-retraining requirement make it suitable for edge deployment in Industrial Internet of Things architectures. The early warning capability, triggered by HI-P as early as Day 4, could enable proactive maintenance scheduling and reduce unplanned downtime. The monotonic degradation trends observed in HI-P suggest prognostic potential for estimating remaining useful life. These results collectively demonstrate that physics-informed multi-sensor fusion with GMM detection and physics-attribution explainability can detect and differentiate multiple co-existing degradation pathways in industrial AODD pump systems without labeled failure data, providing a practical, interpretable, and deployable foundation for predictive maintenance of sludge transfer installations and, more broadly, for industrial systems where labeled fault data are scarce. 

## Figures and Tables

**Table 1:** Sensor configuration.

Sensor ID	Type	Location	Rate (Hz)	Signal
Flow-1	Electromagnetic flowmeter	Inlet line	100	Flow rate
Flow-2	Electromagnetic flowmeter	Outlet line	100	Flow rate
Pneum-1	Pressure transducer	Air supply line	100	Supply pressure
Vib-1	Accelerometer	Inlet manifold	1652	Acceleration
Vib-2	Accelerometer	Outlet manifold	1652	Acceleration

**Table 2:** Dataset summary. All days share an active operating window of approximately 8 h within a 24 h continuous recording period. D1 is excluded from analysis due to a partial operating day and anomalous sensor behaviour during start-up. D2 is used as the sole healthy baseline reference for GMM training.

Label	Period	Active Windows	Status
D1	Pre-baseline	—	Excluded ^†^
D2	Baseline	86,218	Normal (training)
D3	Post-maintenance	86,218	Normal (evaluation) ^‡^
D4	Episode 1	42,112	Anomaly
D5	Episode 1	42,780	Anomaly
D6	Episode 1	39,519	Anomaly
D7	Episode 1 (peak)	39,590	Anomaly
D8	Episode 2	39,590	Anomaly
D9	Episode 2	39,590	Anomaly
D10	Episode 2	39,584	Anomaly
Total (D2–D10)		455,201	

^†^ D1 excluded: partial operating day and anomalous Vib-2 sensor behaviour during the first hour. ^‡^ D3 corresponds to the day on which a confirmed filter press filter change was performed (afternoon). D3 is retained for evaluation but excluded from GMM training to avoid contaminating the healthy reference distribution. Episode 1 (D4–D7) and Episode 2 (D8–D10) labels are assigned post hoc based on the health index trends reported in Section 4.

**Table 3:** Physics-informed health indicators (active-period statistics; D2 = baseline).

Label	Period	$P_{\mathrm{rms}}$	$\kappa_{P}$	$Q_{\mathrm{in},\mathrm{rms}}$	$Q_{\mathrm{out}}/Q_{\mathrm{in}}$	$V_{\mathrm{out},\mathrm{rms}}$	$\kappa_{\mathrm{out}}$	HI-P
D1	Pre-baseline	0.033	−0.76	7.70	1.112	0.067	170,218 ^†^	0.665
D2	Baseline	0.049	−1.44	10.61	1.130	0.046	79	1.000
D3	Post-maint.	0.060	−1.33	8.66	1.124	0.037	178	1.214
D4	Episode 1	0.120	+0.29	11.00	1.167	0.040	79	1.462
D5	Episode 1	0.172	−0.34	12.46	1.180	0.054	60	3.498
D6	Episode 1	0.122	+2.92	8.18	1.201	0.040	69	1.486
D7	Ep.1 peak	0.298	+4.13	6.86	1.211	0.033	123	3.633
D8	Episode 2	0.054	−0.32	11.60	1.156	0.120	24,024	1.092
D9	Episode 2	0.069	−0.44	12.25	1.167	0.090	10,885	1.404
D10	Episode 2	0.091	−0.74	12.54	1.184	0.130	13,254	1.840

^†^ D1 $\kappa_{\mathrm{out}}$ dominated by an isolated electrical spike artefact; excluded from HI-V computation. D3 = day of confirmed filter press filter change.

**Table 4:** Statistical characterisation of degradation episodes. ✓ = Bootstrap 95% CI excludes zero.

Indicator	Comparison	Mean A	Mean B	Ratio	Cohen’s *d*	Bootstrap 95% CI
$P_{\mathrm{rms}}$	Baseline vs. Ep.1	0.054	0.178	3.27×	1.70 (Very Large)	[0.066, 0.204] ✓
$P_{\mathrm{rms}}$	Ep.1 vs. Ep.2	0.178	0.071	0.40×	−1.62 (Very Large)	[−0.188, −0.045] ✓
$V_{\mathrm{out},\mathrm{rms}}$	Baseline vs. Ep.1	0.042	0.042	1.00×	0.00 (Negligible)	[−0.009, 0.009]
$V_{\mathrm{out},\mathrm{rms}}$	Baseline vs. Ep.2	0.042	0.113	2.73×	4.10 (Very Large)	[0.048, 0.089] ✓
$V_{\mathrm{out},\mathrm{rms}}$	Ep.1 vs. Ep.2	0.042	0.113	2.73×	4.81 (Very Large)	[0.050, 0.090] ✓
$\kappa_{\mathrm{out}}$	Ep.1 vs. Ep.2	83	16,055	193.9×	3.61 (Very Large)	—

Comparisons where CI includes zero ($V_{\mathrm{out},\mathrm{rms}}$: Baseline vs. Ep.1) confirm the independence of the two degradation modes.

**Table 5:** GMM anomaly detector performance summary.

Metric	Value	Level
GMM components (*k*, BIC-selected)	6	Model
Training windows (D2 only)	86,218	Model
Total evaluated windows	455,201	Dataset
Window threshold (Youden J)	−4.1828	Model
ROC-AUC	0.8580	Window
PR-AUC	0.9082	Window
KS statistic	0.6543	Window
KS *p*-value	0.00	Window
Accuracy	100.0%	Day
Precision	100.0%	Day
Recall	100.0%	Day
F1 score	1.000	Day
Specificity	100.0%	Day
Episode 1 detection (D4–D7)	4/4 (100%)	Day
Episode 2 detection (D8–D10)	3/3 (100%)	Day

**Table 6:** Sensor ablation study results (GMM, D2-only training, Youden J threshold, 50% day boundary). † = Misses one Episode 2 day.

Configuration	Feat.	ROC-AUC	PR-AUC	Acc.	F1	Ep.1	Ep.2
Pressure only	3	0.8623	0.9207	88.9%	0.923	4/4	2/3 ^†^
Flow only	3	0.7749	0.8015	100%	1.000	4/4	3/3
Vibration only	4	0.7643	0.8258	100%	1.000	4/4	3/3
Pressure + Flow	6	0.8577	0.9093	100%	1.000	4/4	3/3
Pressure + Vibration	7	0.8566	0.9160	88.9%	0.923	4/4	2/3 ^†^
Flow + Vibration	7	0.7839	0.8035	100%	1.000	4/4	3/3
All (Proposed)	10	0.8580	0.9082	100%	1.000	4/4	3/3

**Table 7:** Quantitative comparison of unsupervised anomaly detection methods (this study). All models trained on D2-only baseline (86,218 windows); evaluated on 455,201 windows from nine recording days. Youden J window threshold; 50% day-level decision boundary. † = Misses Episode 2 entirely.

Method	ROC-AUC	PR-AUC	F1	Acc.	Ep.1	Ep.2
Isolation Forest [18]	0.8212	0.8517	1.000	100%	4/4	3/3
One-Class SVM [19]	0.7970	0.8811	0.727	66.7%	4/4	0/3 ^†^
LOF [20]	0.8559	0.9101	1.000	100%	4/4	3/3
Elliptic Envelope [28]	0.7136	0.7203	1.000	100%	4/4	3/3
Proposed GMM (k=6)	0.8580	0.9082	1.000	100%	4/4	3/3

All algorithm implementations follow established practices from recently cited MDPI studies. The experimental results in this table are from our own AODD pump dataset. OC-SVM fails Episode 2 entirely, confirming that the two degradation episodes occupy distinct regions of the feature space.

**Table 8:** Qualitative comparison with related condition-monitoring approaches. ✓✓ = Strong; ✓ = partial; ∼ = limited; × = absent. ^★^ = requires labeled fault data.

Reference	System	Method	No Labels	Physics-Informed	XAI	Real Industrial	Multi-Sensor
Liu et al. [18]	Various	Isolation Forest	✓	×	×	×	×
Zhang et al. [5]	General (survey)	Deep anomaly detection	✓	×	×	∼	∼
Liu et al. [6]	General (survey)	One-class methods (review)	✓	×	×	∼	∼
Wang et al. [21]	Diaphragm pump	Dispersion entropy	✓	×	×	✓	×
Wang et al. [7]	Diaphragm pump	Deep learning fusion ^★^	×	×	×	×	✓
Zhang et al. [9]	Diaphragm pump	PCA-ResNet ^★^	×	×	×	×	×
Mohamed et al. [24]	Rotating machinery	SHAP + ML ^★^	×	×	✓	×	×
Helaoui et al. [1]	Rotating machinery	ML for fault diagnosis	∼	×	×	✓	∼
Proposed (This Work)	AODD Pump (Sludge)	Physics-GMM + Attribution	✓✓	✓✓	✓✓	✓✓	✓✓

The proposed framework is the only approach simultaneously achieving unsupervised operation (no labeled fault data required), physics-informed feature derivation from governing equations, analytically exact physics attribution, real industrial deployment on an AODD sludge-transfer installation, and five-sensor heterogeneous fusion at two sampling rates. Methods marked ^★^ require labeled fault samples unavailable in most industrial deployments. Quantitative comparison of all five competing models on this dataset is reported in Table 7.

## Data Availability

The raw sensor data supporting the conclusions of this article will be made available by the authors upon request.
